# “The injection gives freedom” - An exploration of long-acting injectable HIV treatment acceptance among patients seeking care in two Nairobi tertiary hospitals

**DOI:** 10.1371/journal.pone.0320713

**Published:** 2026-09-30

**Authors:** Duncan Nyukuri, Melanie Abongo, Anastacia Mbithi, Christine Kundu, Kenneth Mutai, Reena Chhotala Shah

**Affiliations:** 1 Department of Medicine, Kenyatta National Hospital, Nairobi, Kenya; 2 Department of Medicine, Aga Khan University Hospital, Nairobi, Kenya; University of Nairobi, KENYA

## Abstract

**Background:**

Long-acting injectable antiretroviral therapy offers an alternative to daily oral HIV treatment, potentially improving adherence and reducing stigma. While its adoption has been successful in some settings, evidence on its acceptability in sub-Saharan Africa, outside clinical trials, remains limited.

**Methods:**

We conducted a mixed-methods study among people living with HIV (PLHIV) in two tertiary hospitals in Nairobi, Kenya—a public facility (Kenyatta National Hospital) and a private facility (Aga Khan University Hospital). A cross-sectional survey of 356 participants assessed awareness, willingness to switch, and predictors of acceptability. In addition, 30 participants took part in three focus group discussions (FGDs) exploring perceptions, concerns, and system-level considerations. Quantitative data were analyzed using descriptive and inferential statistics, while qualitative data were analyzed thematically.

**Results:**

Overall, 72.2% of survey participants indicated a willingness to switch to LAI-ART. In the final adjusted multivariable models, prior awareness of LAI-ART was a powerful independent predictor, nearly tripling a patient’s willingness to switch (aOR = 2.851; 95% CI: 1.742–4.666; p < 0.001). Finding information through online resources (aOR = 2.246; 95% CI: 1.303–3.873; p = 0.004) and missing oral pill doses (aOR = 2.154; 95% CI: 1.266–3.664; p = 0.005) both independently more than doubled a participant’s odds of finding LAI-ART acceptable. Conversely, the enrollment hospital site had no independent statistical effect on acceptability once the analysis controlled for baseline demographics and how often individuals must visit the hospital for care (aOR = 1.188; 95% CI: 0.659–2.141; p = 0.567). Drivers of interest among those willing to switch included reducing pill burden (77.8%), avoiding forgetting doses (55.6%), and improving privacy (45.5%). Despite high baseline acceptability, focus group discussions revealed a state of cautious optimism, driven by significant fears of potential side effects (68.1%), treatment rigidity, and logistical challenges such as travel and clinic access.

**Conclusion:**

These findings suggest that although LAI-ART is widely viewed as a promising treatment option, its uptake will depend on effective patient-centered communication, increased awareness, trust in the intervention, and reliable service delivery. However, successful implementation in Kenya will require more than patient willingness alone. Health system readiness must be a central consideration, with implementation strategies focused on securing dependable drug supply chains, decentralizing access through county-level clinics, and strengthening digital systems to support patient monitoring, retention, and continuity of care.

## Introduction

Long-acting injectable antiretroviral therapy (LAI-ART) offers a promising alternative to the daily pill regimen for treating HIV. Administered every two months, this innovative therapy could simplify treatment, improve patient adherence, and boost retention in care [1,2].

This is especially critical in sub-Saharan Africa (SSA), a region that carries two-thirds of the 40 million people living with HIV worldwide [3]. In Kenya, for example, where 1.4 million people are living with HIV [4], persistent adherence challenges are common due to factors such as pill burden, treatment fatigue, and stigma [5]. These issues are particularly pronounced among adolescents and young people, who often have significantly lower adherence rates than older adults due to heightened stigma and challenges during their transition to adulthood [6]. Consequently, there is an urgent need to explore new and creative strategies to address the unique challenges of the region.

Studies in high-income countries have shown that LAI-ART is as effective as daily oral therapy at maintaining viral suppression, and patient satisfaction is high [7–13]. In the US-based CUSTOMIZE implementation study, participants reported that 92% preferred injectable therapy over their previous oral regimen, and 97% indicated willingness to continue long-acting injections [14]. Despite its promise, real-world implementation presents significant challenges, including concerns about side effects, needle phobia, healthcare provider capacity, and access to healthcare infrastructure capable of supporting this regimen [12,15,16] Crucially, real-world data from outside clinical trials in high-income settings highlight notable discontinuation rates [17,18]. These premature treatment disruptions are frequently driven by injection-site reactions and other adverse events [17,18]. Understanding these implementation barriers is essential, as treatment non-adherence can lead to a resurgence in viral replication and treatment failure.

While there is compelling evidence from North America and Western Europe, there are significant data gaps in sub-Saharan Africa. A 2024 study in Uganda by Zakumumpa et found that 94% of participants in a demonstration project wanted to continue with LAI-ART [19]. However, the results may be biased since participants were already on the treatment. A South African study showed that while only 12% of adolescents and young people with HIV initially preferred LAI-ART, this preference increased to 66% among those who faced multiple adherence challenges like stigma and pill burden [20]. Research from the Dominican Republic, Tanzania, and a small 2018 study in Kenya also showed broad acceptance of LAI-ART, driven by the desire for reduced stigma and greater privacy [16]. A recent scoping review also found high acceptability for LAI-ART in sub-Saharan Africa, ranging from 63% to 98% [21]. Still, most of these studies were conducted among specific populations, like those in clinical trials or high-risk, HIV-negative individuals, which limits their broader applicability.

This study builds on prior work by examining the acceptability of LAI-ART among a more general population of PLHIV receiving care in two tertiary hospitals in Nairobi—one public and one private. Using a mixed-methods approach grounded in the Consolidated Framework for Implementation Research (CFIR) [22], we explore perspectives of patients currently on oral ART who have not yet used injectables. By addressing both individual and system-level factors, this study provides actionable insights to support equitable LAI-ART implementation in Kenya and similar settings across SSA.

## Materials and methods

### Study design

This was an exploratory mixed-methods study using a convergent parallel design, conducted to assess the acceptability of long-acting injectable antiretroviral therapy among people living with HIV in Kenya. A mixed-methods design was chosen to provide both breadth and depth in understanding LAI-ART acceptability. Quantitative data captured the extent and predictors of willingness to switch, while qualitative findings explained the underlying motivations, concerns, and contextual factors influencing these attitudes. Combining both approaches allowed for a more comprehensive understanding of patient perspectives than either method alone. Importantly, this was not a clinical trial; participants were not enrolled into any intervention arm, and no medications were administered.

### Study site and participants

The study was conducted at two tertiary hospitals in Nairobi, Kenya: Aga Khan University Hospital (AKUH) and Kenyatta National Hospital (KNH). AKUH, a private not-for-profit institution, is a referral center offering specialized care. KNH is the largest public hospital in Kenya and a teaching facility for the University of Nairobi, supporting over 8,000 PLHIV in outpatient care.

The study was conducted at AKUH between December 14, 2023, and January 12, 2024, and at KNH between May 21, 2024, and July 9, 2024. Eligible participants were adults (≥18 years), currently receiving oral ART, and enrolled in HIV outpatient care at either facility. Participants were excluded if they had cognitive impairments, were acutely unwell, or could not communicate in English or Kiswahili.

### Sampling and recruitment

Quantitative participants were selected using systematic random sampling from outpatient clinic attendance logs to achieve a representative sample. The sample size was calculated using Cochran’s formula for cross sectional studies [23] with a 95% confidence level and a 5% margin of error. The acceptability rate of 66%, which was used for the calculation, was derived from a South African study by Toska et al [20]. This study explored the acceptability of long-acting injectable LAI-ART among adolescents and young people living with HIV (AYLHIV) who were currently on oral therapy, and this prevalence was chosen because it yielded the largest sample size. This resulted in a minimum required sample size of 345 participants.

Participants who consented to participate in the survey were approached during clinic visits and invited to take part in focus group discussions. The participants were selected via purposive sampling based on gender, age, and clinic site to capture diverse perspectives.

### Data collection procedures

#### Quantitative data.

The Consolidated Framework for Implementation Research (CFIR) guided the development of survey tools and focus group guides, and later informed the interpretation of qualitative themes to explore multi-level barriers and facilitators to LAI-ART adoption (20). A structured questionnaire was used to collect data on demographics, knowledge of LAI-ART, and attitudes toward injectable regimens. The data collection tool used had been developed and validated by Simoni et al [24] in a similar study conducted in Tanzania and the Dominican, and it was also based on CFIR constructs. Data were collected in person or via REDCap online survey links.

#### Qualitative data - Focus group discussions (FGDs).

Three FGDs were conducted, with 10 participants per FGD – the recommended number consisting of between 6 and 12 participants per group [25] – to foster open discussion. Discussions were held in private rooms at each study site and moderated by M.A, an experienced qualitative researcher in conducting FGDs, with no clinical relationship to participants. A semi-structured discussion guide covered themes such as LAI-ART awareness, perceived benefits and drawbacks, implementation concerns, and facility readiness. Sessions were audio-recorded with consent; field notes were not taken. Discussions continued until thematic saturation was reached. Thematic saturation was assessed by the point at which no new themes emerged during subsequent discussions

### Data analysis

#### Quantitative analysis.

Survey data were analyzed using SPSS version 25. Both descriptive and inferential analyses were conducted. Participant characteristics were summarized separately for each study site (KNH and AKUH) and for the combined study population. Categorical variables, including demographic characteristics, social history, and medical history, were presented as frequencies and percentages.

Inferential analyses were undertaken in a stepwise manner. Initially, bivariate cross-tabulations were used to examine the distribution of LAI-ART acceptability across participant characteristics. Statistical significance was assessed using Pearson’s chi-squared (χ²) test of independence or Fisher’s exact test where appropriate. To quantify the magnitude and direction of these associations and support variable selection for multivariable analysis, univariable logistic regression was performed to estimate crude odds ratios (cORs) and their corresponding 95% confidence intervals (CIs).

Variables associated with the outcome at a p-value of <0.20 in the univariable analysis were considered for inclusion in multivariable models. Age, gender, and study site were retained in all multivariable models regardless of statistical significance because of their established clinical and epidemiological relevance.

Multivariable logistic regression was subsequently performed using an exposure-specific hierarchical modelling strategy. Four separate models were constructed to minimize intermediate-variable and overadjustment bias while allowing a more nuanced assessment of the factors associated with LAI-ART acceptability.

Model 1 examined socio-demographic characteristics, including age, gender, education level, and enrolment hospital. Model 2 assessed the association between prior awareness of long-acting injectables and acceptability while adjusting for socio-demographic characteristics. Model 3 evaluated specific information sources, including online platforms, healthcare providers, and peer support groups, while controlling for socio-demographic factors. Model 4 examined clinical and treatment-related factors, including frequency of hospital visits, self-reported adherence, missed doses, and perceived pill burden, with adjustment for socio-demographic characteristics.

Adjusted odds ratios (aORs) with 95% confidence intervals were reported for all multivariable analyses. Statistical significance was defined as a two-sided p-value < 0.05. Multicollinearity was assessed by examining coefficient stability and standard errors to ensure that highly correlated variables did not adversely affect model estimates.

#### Qualitative analysis.

Audio recordings from focus group discussions were transcribed verbatim. For each transcript, structured summary templates were completed using pre-specified domains derived from the CFIR (S1 Fig). Two independent coders (DN and CK) reviewed each transcript to identify blocks of text corresponding to relevant CFIR constructs. Data were aggregated across participants to facilitate comparison within and across domains. The two researchers discussed coding decisions, resolved discrepancies through consensus, and agreed on emergent themes. We did not return transcripts to participants for feedback or validation due to logistical and confidentiality constraints. However, the use of a structured summary template, independent dual coding, and consensus discussions enhanced the reliability and rigor of the analysis.

#### Integration of findings.

Quantitative and qualitative data were analyzed independently before being integrated during interpretation through triangulation to compare and explain convergent and divergent findings. The guidelines for Good Reporting of a Mixed Methods Study (GRAMMS), as shown in S1 Table, was used in integrating the findings of the qualitative and quantitative arms of the study (21). Results were jointly displayed to enhance understanding of complex, multi-level factors influencing LAI-ART acceptability.

### Ethical statement

Ethical approval for this study was obtained from both the Kenyatta National Hospital–University of Nairobi Ethics and Research Committee (Approval No. P816/10/2023) and the Aga Khan University Institutional Research and Ethics Committee (Approval No. 2023/ISERC-50(v3)). All participants provided written informed consent prior to enrollment, after receiving detailed explanations of the study’s objectives, procedures, potential risks, and measures taken to protect confidentiality.

Participation was entirely voluntary, and refusal or withdrawal did not affect participants’ access to clinical care. Data were anonymized at the point of collection, and no personal identifiers were linked to survey or focus group responses. Focus group discussions were conducted in private rooms to safeguard participants’ privacy and minimize stigma-related risks. A trained, experienced and independent moderator facilitated the discussions in a manner sensitive to participants’ experiences. All findings are reported in aggregate to ensure that no individual can be identified.

## Results

A total of 356 individuals living with HIV participated in the study, with a substantial majority receiving care within the public healthcare sector at Kenyatta National Hospital (KNH, 77.2%) compared to the private sector at the Aga Khan University Hospital (AKUH, 22.8%). Taken as a whole, the study population was predominantly female (59.0%), and the largest age bracket comprised individuals in their middle adult years between 40 and 49 years of age (32.3%). The vast majority of participants across both facilities were balancing familial and financial responsibilities; 61.2% were married, 81.7% were parents/guardians raising children, and 81.7% were actively employed. Social histories revealed relatively low rates of recreational substance use across the entire cohort, with only 5.6% reporting active cigarette smoking and 1.1% reporting regular recreational drug use. A comprehensive breakdown of these baseline characteristics is detailed in Table 1.

**Table 1:** Socio-demographics characteristics.

Characteristics		Site					
		KNH		AKUH		Total	
		N	%	N	%	N	%
Age group	20-29	29	10.5	*	*	*	*
	30-39	83	30.2	10	12.3	93	26.1
	40-49	86	31.3	29	35.8	115	32.3
	50-59	66	24.0	30	37.0	96	27.0
	60+	11	4.0	10	12.3	21	5.9
Gender	Female	174	63.3	36	44.4	210	59.0
	Male	101	36.7	45	55.6	146	41.0
Marital status	Married	168	61.1	50	61.7	218	61.2
	Separated/Divorced	16	5.8	12	14.8	28	7.9
	Single	71	25.8	16	19.8	87	24.4
	Widow(Er)	20	7.3	3	3.7	23	6.5
Living arrangement	Alone	58	21.1	29	35.8	87	24.4
	As A Couple	15	5.5	10	12.3	25	7.0
	With Family	202	73.5	42	51.9	244	68.5
Has children	No	46	16.7	19	23.5	65	18.3
	Yes	229	83.3	62	76.5	291	81.7
Employment status	No	51	18.5	14	17.3	65	18.3
	Yes	224	81.5	67	82.7	291	81.7
Cigarette use	No	256	93.1	80	98.8	336	94.4
	Yes	19	6.9	1	1.2	20	5.6
Alcohol use	No	223	81.1	59	72.8	282	79.2
	Yes	52	18.9	22	27.2	74	20.8
Recreational drugs use	No	272	98.9	80	98.8	352	98.9
	Yes	3	1.1	1	1.2	4	1.1

*Cell suppressed (N < 5) to protect participant anonymity.

When we looked closer at the two hospital populations, several clear socio-demographic differences emerged that reflect the distinct socio-economic landscapes of public and private healthcare in Nairobi. The private cohort at AKUH was predominantly male (55.6% vs. 36.7% at KNH) and older, with more than a third of its patients sitting in the 50–59 age bracket (37.0% vs. 24.0% at KNH). Living situations also differed significantly between the two groups. While nearly three-quarters of KNH patients shared living arrangements with their families (73.5%), over a third of AKUH patients lived alone (35.8% vs. 21.1% at KNH).

The clinical profiles and treatment experiences of the participants, summarized in Table 2, mirror these demographic variations. The higher burden of age-associated metabolic comorbidities observed in AKUH cohort likely reflects its older average age. Specifically, AKUH patients experienced nearly quadruple the rate of diabetes mellitus (14.8% vs. 4.0% at KNH) and close to quadruple the prevalence of high cholesterol (12.3% vs. 3.3% at KNH). They also had significantly longer histories of living with HIV, with roughly half having managed their diagnosis for over a decade (49.4% vs. 29.1% at KNH). This extensive treatment history likely explains why nearly half of the AKUH patients had switched antiretroviral therapy (ART) regimens in the past, primarily due to cumulative drug tolerance issues (35.8% vs. 8.4% at KNH).

**Table 2:** Medical history.

Characteristics		Site					
		KNH		AKUH		Total	
		N	%	N	%	N	%
Hypertension	No	233	84.7	64	79.0	297	83.4
	Yes	42	15.3	17	21.0	59	16.6
Diabetes	No	264	96.0	69	85.2	333	93.5
	Yes	11	4.0	12	14.8	23	6.5
High cholesterol	No	266	96.7	71	87.7	337	94.7
	Yes	9	3.3	10	12.3	19	5.3
Psychiatric disorder/depression	No	243	88.4	79	97.5	322	90.4
	Yes	32	11.6	2	2.5	34	9.6
Duration HIV Treatment	1-5 Years	80	29.1	20	24.7	100	28.1
	5-10 Years	63	22.9	15	18.5	78	21.9
	I Prefer Not To Say	31	11.3	0	0.0	31	8.7
	Less Than 1 Year	21	7.6	6	7.4	27	7.6
	More Than 10 Years	80	29.1	40	49.4	120	33.7
Currently taking ART	More Than Twice/Day	1	0.4	0	0.0	1	0.3
	Once/Day	263	95.6	78	96.3	341	95.8
	Twice/Day	11	4.0	3	3.7	14	3.9
ART treatment perception	Complicated	23	8.4	6	7.4	29	8.1
	Simple	252	91.6	74	91.4	326	91.6
	Unmanageable	0	0.0	1	1.2	1	0.3
Adverse effects from ART	Never	153	55.6	49	60.5	202	56.7
	Major	2	0.7	4	4.9	6	1.7
	Minor	120	43.6	28	34.6	148	41.6
ART treatment tolerance	No	40	14.5	8	9.9	48	13.5
	Yes	235	85.5	73	90.1	308	86.5
Missed ART doses	Never	109	39.6	64	79.0	173	48.6
	Regularly	6	2.2	0	0.0	6	1.7
	Sometimes	160	58.2	17	21.0	177	49.7
Hospital visit frequency	Every 2–3 Months	168	61.1	74	91.4	242	68.0
	Every 6 Months	107	38.9	7	8.6	114	32.0
Partner aware of HIV status	I Prefer Not To Say	17	6.2	5	6.2	22	6.2
	No	96	34.9	23	28.4	119	33.4
	Yes	162	58.9	53	65.4	215	60.4
Family aware of HIV status	I Prefer Not To Say	15	5.5	1	1.2	16	4.5
	No	127	46.2	48	59.3	175	49.2
	Yes	133	48.4	32	39.5	165	46.3
Friends aware of HIV status	I Prefer Not To Say	15	5.5	3	3.7	18	5.1
	No	224	81.5	70	86.4	294	82.6
	Yes	36	13.1	8	9.9	44	12.4
Colleagues aware of HIV status	I Prefer Not To Say	14	5.1	4	4.9	18	5.1
	No	247	89.8	68	84.0	315	88.5
	Yes	14	5.1	9	11.1	23	6.5
ART Change Effectiveness	I Don’T Know	3	1.1	6	7.4	9	2.5
	No	231	84.0	63	77.8	294	82.6
	Yes	41	14.9	12	14.8	53	14.9
ART change Tolerance	I Don’T Know	3	1.1	6	7.4	9	2.5
	No	249	90.5	46	56.8	295	82.9
	Yes	23	8.4	29	35.8	52	14.6
ART change Adherence	I Don’T Know	1	0.4	7	8.6	8	2.2
	No	267	97.1	74	91.4	341	95.8
	Yes	7	2.5	0	0.0	7	2.0

In contrast, the public sector cohort at KNH presented a very different set of daily clinical realities. KNH participants reported a noticeably higher burden of mental health struggles, with a 11.6% prevalence of documented psychiatric disorders or depression compared to just 2.5% among their private-sector counterparts. Most strikingly, their day-to-day experiences maintaining strict adherence to daily oral medication revealed substantial structural and behavioral vulnerabilities. A striking 60.4% of KNH patients acknowledged that they missed their daily oral pill doses either sometimes or regularly, whereas an overwhelming majority of AKUH patients reported perfect adherence (79.0% never missed a dose). These daily struggles persisted despite the fact that 91.6% of the entire study population described their current once-daily oral pill regimen as conceptually “simple” to understand. Finally, the structural rhythm of accessing care differed deeply between the institutions: 91.4% of AKUH patients had routine clinic visits scheduled closely every 2–3 months, whereas nearly 40% of KNH patients spaced their facility interactions further apart, visiting only once every 6 months.

### Knowledge and acceptability of LAI-ART

As shown in Table 3, 65.2% of participants had prior knowledge of LAI-ART, most commonly from a healthcare provider (37.4%) or online resources (36.0%). Acceptability was high, with 72.2% expressing willingness to use LAI-ART. However, 17.4% reported they lacked sufficient information to decide, and 8.4% indicated they would not accept it.

**Table 3:** LAI-ART awareness & acceptability.

Characteristic		Site					
		KNH		AKUH		Total	
		N	%	N	%	N	%
Aware of LAI-ART	No	99	36.0	25	30.9	124	34.8
	Yes	176	64.0	56	69.1	232	65.2
Source of information	Healthcare provider	102	37.1	31	38.3	133	37.4
	Support group	59	21.5	11	13.6	70	19.7
	Online resources	87	31.6	41	50.6	128	36.0
	Other	1	0.4	2	2.5	3	0.8
Acceptability of LAI-ART	I Am Not Sure	2	0.7	5	6.2	7	2.0
	I Don’T Have Enough Information	42	15.3	20	24.7	62	17.4
	No	25	9.1	5	6.2	30	8.4
	Yes	206	74.9	51	63.0	257	72.2

Site differences were observed. Acceptability was higher at KNH (74.9%) compared to AKUH (63.0%). KNH participants were more likely to learn about LAI-ART from healthcare providers, whereas AKUH participants more frequently cited online sources.

### Factors associated with acceptability of LAI-ART

To evaluate potential predictors of willingness to switch to a long-acting injectable regimen, a bivariate cross-tabulation screening was performed across all demographic, social, and clinical parameters collected in the study. Associations were evaluated using Chi-square tests of independence, with Fisher’s exact tests applied in instances where individual cell counts dropped below five. To quantify the unadjusted strength and direction of these baseline relationships, univariable logistic regression was utilized to calculate crude odds ratios (cOR). The complete statistical breakdowns, alongside these crude odds ratios with their 95% confidence intervals, are consolidated in S1 Table.

When we examined the socio-demographic factors, the primary clinic site where a patient receives care showed a statistically significant crude association with willingness to switch. Acceptability was notably higher among patients at KNH, compared to AKUH (74.9% vs. 63.0%; p = 0.035). Age also showed a clear generational pattern, with younger cohorts demonstrating the highest level of interest. Acceptability peaked at 80.6% among young adults aged 20–29 years and gradually declined to 57.1% among patients aged 60 years and older, although this difference did not reach statistical significance (p = 0.141). Other background demographics like gender (p = 0.124), marital status (p = 0.142), employment status (p = 0.230), and living arrangements (p = 0.069) did not show significant crude associations with acceptability. Similarly, social history factors like cigarette use (p = 0.822) and alcohol use (p = 0.902) had no notable impact. Looking at medical history and comorbidities, specific conditions like hypertension (p = 0.897), diabetes (p = 0.502), high cholesterol (p = 0.499), and psychiatric disorders (p = 0.323) did not significantly alter participant willingness to switch. Furthermore, historical treatment milestones, such as the total time since HIV infection (p = 0.507), duration of HIV treatment (p = 0.796), and whether a patient had previously changed regimens due to effectiveness (p = 0.806) or tolerance (p = 0.606), showed no meaningful baseline relationship with acceptability. Patterns of HIV status disclosure also had no apparent influence on attitudes toward LAI-ART. Whether a patient’s HIV status was known by a partner (p = 0.593), family member (p = 0.460), friend (p = 0.422), or colleague (p = 0.210), acceptability remained unchanged. In contrast, a patient’s daily relationship with their current oral pill routine and their access to information were highly significant predictors of treatment acceptability. Patients who felt their current daily regimen was structurally “complicated” were significantly more likely to find the injectable acceptable than those who viewed their routine as simple (p = 0.023). Likewise, patients who reported side effects showed higher willingness to switch (p = 0.035). Most notably, patients who acknowledged missing daily oral doses either sometimes or regularly demonstrated a highly significant crude preference for switching over to LAI-ART (p < 0.001). Finally, prior awareness of LAI-ART was strongly associated with greater acceptability (p < 0.001). Analysis of sources of information showed that participants who received information about LAI-ART from online sources were significantly more likely to accept the treatment option (p = 0.009). Exposure through peer support groups showed a similar trend and was associated with higher acceptability, although this relationship fell just short of statistical significance (p = 0.054). In contrast, learning about LAI-ART directly from healthcare providers was not associated with differences in acceptability, with attitudes distributed similarly across groups (p = 0.465).

### Multivariable predictors of LAI-ART acceptability

To determine the independent predictors of long-acting injectable antiretroviral therapy acceptability and control for confounding without introducing intermediate-variable bias, we built four separate, exposure-specific multivariable binary logistic regression models. Following epidemiological principles, core baseline demographic factors (facility site, age group, and gender) were forced into all models to control for residual confounding, regardless of their statistical significance in the bivariate screening. A global “First” category setting was used for all variables in SPSS 25. This established our baseline reference comparison groups as the Aga Khan University Hospital (AKUH), female gender, the 20–29 age group, an absence of prior informational exposure (“No”), a current treatment perception of “Complicated,” and an absence of daily oral treatment barriers (“No”). The adjusted odds ratios (aOR), 95% confidence intervals (CI), and exact p-values for all four models are summarized in Table 4.

**Table 4:** Multivariable logistic regression models predicting the acceptability of LAI-ART (N = 356).

Predictor / Exposure Variable	Model 1: Site, aOR (95% CI); p-value	Model 2: Awareness, aOR (95% CI); p-value	Model 3: Information source, aOR (95% CI); p-value	Model 4: Clinical Experience, aOR (95% CI); p-value
**Site** *(Ref:* *AKUH*)				
KNH	1.474 (0.846–2.568); p = 0.171	1.605 (0.902–2.857); p = 0.108	1.712 (0.958–3.058); p = 0.069	1.188 (0.659–2.141); p = 0.567
**Gender** *(Ref: Female)*				
Male	0.725 (0.447–1.175); p = 0.191	0.740 (0.451–1.215); p = 0.234	0.747 (0.456–1.226); p = 0.249	0.713 (0.434–1.169); p = 0.180
**Age Group** *(Ref: 20–29)*				
30–39 years	0.866 (0.311–2.411); p = 0.782	0.887 (0.311–2.530); p = 0.823	0.877 (0.310–2.482); p = 0.805	0.938 (0.327–2.691); p = 0.906
40–49 years	0.506 (0.189–1.354); p = 0.175	0.497 (0.181–1.364); p = 0.175	0.494 (0.182–1.346); p = 0.168	0.575 (0.209–1.581); p = 0.284
50–59 years	0.724 (0.263–1.994); p = 0.532	0.773 (0.274–2.177); p = 0.625	0.772 (0.276–2.158); p = 0.622	0.965 (0.337–2.762); p = 0.948
60 + years	0.382 (0.107–1.362); p = 0.138	0.374 (0.101–1.379); p = 0.139	0.406 (0.112–1.474); p = 0.171	0.580 (0.156–2.155); p = 0.416
**Prior Awareness** *(Ref: No)*				
Yes	—	**2.851 (1.742–4.666); p < 0.001**	—	—
**Online Resources** *(Ref: No)*				
Yes	—	—	**2.246 (1.303–3.873); p = 0.004**	—
**Support Groups** *(Ref: No)*				
Yes	—	—	1.706 (0.865–3.363); p = 0.123	—
**ART** **Perception** *(Ref: Complicated)*				
Simple	—	—	—	0.354 (0.101–1.242); p = 0.105
**Adverse Effects** *(Ref: No)*				
Yes	—	—	—	1.388 (0.829–2.322); p = 0.212
**Missed** **ART** **Doses** *(Ref: No)*				
Yes	—	—	—	**2.154 (1.266–3.664); p = 0.005**

Model 1 tested the total independent effect of the enrollment facility site on treatment acceptability, intentionally leaving out downstream informational variables that could act as intermediates. Although our bivariate analysis showed that patients at KNH had a higher unadjusted rate of acceptability compared to AKUH (p = 0.035), this difference disappeared after multivariable control. When adjusting for baseline age and gender, enrollment at KNH was not significantly associated with acceptability (aOR = 1.474; 95% CI: 0.846–2.568; p = 0.171). This shows that the initial differences between the hospitals were actually driven by the different demographic backgrounds of their patient populations.

Model 2 looked at the total independent impact of general prior awareness about the injectable regimen. The multivariable model showed that prior knowledge was a powerful independent predictor of acceptability. Patients who already knew about LAI-ART had nearly triple the odds of finding it acceptable compared to those with no prior knowledge (aOR = 2.851; 95% CI: 1.742–4.666; p < 0.001). Within this model, the independent effects of the hospital site (p = 0.108) and male gender (p = 0.234) remained non-significant.

Model 3 included sources of information that were significant (online resources, p = 0.009) or marginally significant (peer support groups, p = 0.054) in the multivariate analysis. Finding information through online resources remained a strong independent predictor, more than doubling the odds of treatment acceptability (aOR = 2.246; 95% CI: 1.303–3.873; p = 0.004). Sourcing information from support groups trended positively but did not reach statistical significance (aOR = 1.706; 95% CI: 0.865–3.363; p = 0.123).

Model 4 evaluated the total independent effect of patients’ current oral ART treatment experiences on their willingness to switch to the injectable format. This model simultaneously entered treatment perception, a history of side effects, and patterns of missed doses. Patients who reported missing daily oral doses had more than double the adjusted odds of finding the long-acting injectable acceptable compared to those who never missed a dose (aOR = 2.154; 95% CI: 1.266–3.664; p = 0.005). Also. patients who experienced side effects from oral therapy had a 39% higher preference of LAIART compared to those who did not report any side effects, but this difference was not statistically significant (aOR = 1.388; 95% CI: 0.829–2.322; p = 0.212). Similarly, perceiving the current oral regimen as “Simple” was associated with lower odds of wanting to switch compared to those who viewed it as complicated, but this difference was not statistically significant (aOR = 0.354; 95% CI: 0.101–1.242; p = 0.105).

### Preferences, advantages & disadvantages of LAI-ART

Among those willing to use LAI-ART, the majority favored longer injection intervals, with three months (27.2%) and six months (35.8%) being the most common choices (S1 Table). Preferences varied by site, with AKUH participants more likely to prefer two-monthly dosing, while KNH participants leaned toward six-monthly schedules.

The most frequently cited advantages were the ability to stop daily pill-taking (77.8%), to avoid forgetting doses (55.6%), and to reduce daily focus on HIV (50.2%) (S1 Table)**.** Other benefits included improved privacy (45.5%) and the ability to “forget the disease” (37.0%).

The most common disadvantages were fear of side effects (68.1%), fear of injections (28.0%), and concerns about being treated as “guinea pigs” (17.9%) (S1 Table)**.** Site differences emerged: AKUH participants were more likely to cite effectiveness and dose adherence as advantages, but also more frequently reported fear of side effects. In contrast, KNH participants more often valued privacy-related benefits and reported fear of injections.

### Qualitative findings and integrated analysis

A summary of emergent themes and sub-themes across CFIR domains, along with illustrative quotes, is presented in Table 5.

**Table 5:** Emergent themes and sub-themes from qualitative analysis based on the CFIR.

CFIR Domain	Theme	Subtheme	Illustrative Quote(s)
Individual Characteristics	Knowledge and Beliefs	Information gaps on side effects and efficacy	*“I wanted to ask…* *Maybe you can tell us if they have any side effects so that we get to know what to expect.”* **(FGD3 R1)**
		Positive perception of convenience and freedom	*“I heard they are coming to Kenya, and I prefer the injectable over oral because oral can be inconvenient in case you have visitors and you* *fear taking the tablet. The injection gives freedom.”* **(FGD1 R2)**
		Concerns about medical and fertility impacts	*“What if they inject, and at the end of the day, I won’t be able to be productive?”* **(FGD2 R9)**
	Individual Stage of Change	Varied readiness for adoption	*“If I’m honest, I won’t take injectable. I will take the tablets until it has been tested on others.”* **(FGD3 R7)** *“Yes. It’ll be better to get injection, yes, than the tabs”* **(FGD1 R6).**
	Personal Motivation	Desire to overcome stigma and pill burden	*“I’m saying even that injection, if it works well, it will be an advantage to the students, those who are in the schools. It will keep that secret; there won’t be any trauma then.”* **(FGD2 R7)** *“It’s been an embarrassment I’ve gotten from taking the tablets. I’ve gotten the greatest shock. In as much as it has helped me, outside there when you get sick a little bit, people know.”* **(FGD 2R4)**
Intervention Characteristics	Perceived Advantages	Convenience and improved adherence	*“I won’t forget, and it will help me keep time.”* **(FGD3 R7)***“The injectables are good because you don’t have to worry until your next appointment.”* **(FGD3 R1)**
		Reduced pill burden	*“I prefer injections because swallowing tablets has always been a problem for me. If I had something like an injection, I will go for that than a tablet.”* **(FGD1 R5)**
		Fear of irreversible side effects	*“Suppose you get the side effects, is it reversible or will it continue that way? Is there any other back up? What can one do to remove the medicine which is in your body already?”* **(FGD1 R7)** *“Will the injection affect my sleep or cause insomnia?”* **(FGD 3R4).** *“You know we must know the side effects.”* **(FGD 2 R1)** *“The body will get used to the side effects.”* **(FGD 3 R9).**
	Perceived Disadvantages	Logistical inflexibility compared to oral ART	*“If I am injected in Kenyatta* *and I travel to Western Kenya, what will I do if I can’t come back for my next injection?”* **(FGD 2 R8).** *“The disadvantage of injections is that with tablets you can carry extras and reschedule if you miss.”* **(FGD 3 R4).** *“What I would like to know is if I skip the dates for my injection and I come two days later what will happen?”* **(FGD1 R1)**
Inner Setting	Healthcare Delivery System	Need for continued support (reminders, education)	*“There was a reminder—they should use it again during injectables to help me to know dates and time. That would be the biggest support”* **(FGD3 R5)** *“You know, when you’re at* *this point when you’re doing transitioning, a lot happens. There are so many things like the side effects and other things. So, we need to have, uh, follow-up. Phones and follow-up”* **(FGD2 R7)** *“The apps like the one we used to have that give information like weight, BMI, remind of dates, and also as we start you give us more clinics for feedback and more information”* **(FGD3 R8).**
		Concerns about resource availability and equity	*“Will it be available all the time and continuous?”* **(FGD3 R5)** *“Also, this one, when it comes and let’s say it’s been accepted, it doesn’t have any side effects, does it mean every county we will be going? Just like how we get our ARVs even at the county levels”* **(FGD2 R5).**
		Safety and Monitoring	*“Before they inject, do they test* *your blood??”* **(FGD 1 R5).** *“Is it for everyone like, children, adults, those who have other conditions like pressure, diabetes?”* **(FGD3 R8)** *“We need to know the manufacturer.”* **(FGD3 R3).**
Outer Setting	Societal Context	Overcoming stigma from public pill-taking	*“For me injectable is okay, I am a housekeeper, I stay with my employer and our employers they don’t like people living with HIV, they think you might affect their children. When they realize you have the tablets, they will just throw you out and add more stigma, but for injectables I will be comfortable and it will help reduce stigmatization.”* **(FGD3 R1)** *“These young ones, they feel shy walking with the drugs and they fear using them in front of people…* *we would prefer the injectable ones that one won’t feel shy anymore and you can go anywhere with it.,.”* **(FGD 2 R3)** *“Sometimes you hide yourself when taking pills because people start asking ‘Why is she taking the pills?’ With injections, nobody will know why I am going for injection.”* **(FGD3 R5)**
		Impact of accidental disclosure on family	*“One time when my brother left and collected my belonging in my house and found my medicine, he left, took alcohol and came back. I felt like killing myself... Remember he is a brother and right now he has told everyone whom they are drinking alcohol with. Let me tell you, there is nothing as painful as that.”* **(FGD 2 R4)**

### Integration of findings

#### Convergence: Reinforcing survey data.

**The Burden of Pills and Stigma:** Survey data showed limited disclosure of HIV status: only 12.4% had disclosed to friends and 6.5% to colleagues. Also, nearly half (45.5%) cited the ability to hide treatment for privacy as a perceived advantage of LAI-ART. FGDs contextualized this, with participants describing stigma and discretion as strong motivations:

*“I prefer the injectable over oral because oral can be inconvenient in case you have visitors and you fear taking the tablet. The injection gives freedom.”* (FGD1, R2)

*“For me injectable is okay, I am a housekeeper…our employers don’t like people living with HIV. When they realize you have the tablets, they will just throw you out and add more stigma, but for injectables I will be comfortable and it will help reduce stigmatization.”* (FGD3, R1)

**Fear and Distrust of New Treatments:** Quantitatively, 68.1% cited fear of side effects, 28.0% feared injections, and 17.9% worried about being treated as “guinea pigs.” This lines up directly with the non-significant multivariable findings for oral side effects in Model 4 (p = 0.212), showing that current side effects are not what drives acceptability; rather, it is the fear of future, irreversible side effects from the injectable that creates hesitation. FGDs elaborated that the main concern was the irreversibility of long-acting drugs and the desire to see them proven safe first:

*“If it has already been injected into the body and it has to stay for 3 months but you are very reactive, it will be difficult to stop—for example, allergies.”* (FGD2, R1)

*“If I’m honest, I won’t take injectable. I will take the tablets until it has been tested on others.”* (FGD3, R7)

**Adherence Motivation:** In Model 4, objective clinical experiences emerged as a dominant independent driver of willingness to switch. Specifically, patients who reported missing their daily oral doses either sometimes or regularly had more than double the adjusted odds of finding LAI-ART acceptable compared to those with perfect adherence (aOR = 2.154, p = 0.005). The qualitative narratives perfectly matched this quantitative reality, highlighting that daily pill fatigue makes a long-acting option highly desirable:

*“I prefer injections because swallowing tablets has always been a problem for me. If I had something like an injection, I will go for that than a tablet.”* (FGD 1R5.)

*“The injectables are good because you don’t have to worry until your next appointment.”* **(FGD 3 R1***).*

### Divergence and nuance: Highlighting tensions

**Cautious Optimism vs. High Acceptability:** Although surveys showed 72.2% acceptability, qualitative data revealed this represented a form of cautious optimism rather than unconditional support. Many participants preferred a trial period to assess safety before committing long-term:

*“I will take the injection for three months to weigh the side effects, then commit for a year if it reacts positively.”* (FGD3, R1)

**Convenience vs. Flexibility:** Survey responses suggested broad willingness to use LAI-ART, but FGDs revealed concerns about reduced flexibility compared to oral ART, particularly when travel or missed appointments were involved:

*“The disadvantage of injections is that with tablets you can carry extras and reschedule if you miss.”* (FGD3, R4)

*“If I am injected in Kenyatta and I travel to Western Kenya, what will I do if I can’t come back for my next injection?”* (FGD2, R8)

**System-Level Demands Beyond the Injection:** Our final multivariable models isolated individual-level behaviors like information channels and adherence challenges as independent predictors of acceptability. However, the qualitative analysis brought to light systemic health infrastructure demands that a standard survey cannot easily capture. Participants noted that individual willingness means very little if the broader health sector is not prepared to guarantee decentralized access, drug continuity, and reliable follow-up systems:

*“You know, when you’re at this point when you’re doing transitioning, a lot happens… So we need to have follow-up. Phones and follow-up.”* (FGD2)

*“…does it mean every county we will be going? Just like how we get our ARVs even at the county levels?”* (FGD2, R5)

*“Before they inject, do they test your blood?”* (FGD1, R5)

## Discussion

The main objective of this study was to assess the acceptability of long-acting injectable antiretroviral therapy (LAI-ART) and the socio-demographic and clinical factors associated with it among people living with HIV (PLHIV) receiving care at two tertiary hospitals in Nairobi, Kenya.

Overall, 72% of participants indicated a willingness to transition to an injectable regimen. This interest was largely driven by the prospect of reducing the burden of daily pill-taking, minimizing the risk of inadvertent HIV status disclosure, and overcoming challenges with adherence. However, enthusiasm for LAI-ART was not without reservations. Many participants expressed concerns about potential side effects, the inflexibility of scheduled clinic visits, and difficulties accessing injection services within their local communities.

In the exposure-specific multivariable models, the strongest independent predictors of acceptability were prior awareness of LAI-ART, obtaining information from online sources, and experiencing adherence difficulties such as missing daily oral doses. By contrast, the apparent association between care setting (public versus private sector) and acceptability disappeared after adjustment for confounders. This suggests that the initial difference between institutions was explained by underlying patient characteristics, particularly age, gender, and the extent to which individuals struggled with daily oral therapy (24–26)

When comparing our 72.2% acceptability rate with international data, it is important to distinguish between pre-implementation surveys and active demonstration projects. In high-income countries, where cabotegravir/rilpivirine is already integrated into routine care, uptake and satisfaction rates often exceed 90%, as shown in the US-based CUSTOMIZE study [14]. Similarly, a 2024 demonstration project in Uganda reported a 94% continuation preference [19]. However, direct comparison with these studies is misleading because participants in demonstration trials have already accepted injections, received intensive counseling, and benefited from subsidized logistics. Our study reflects a routine outpatient population in both public and private sectors before formal rollout of LAI-ART. In that context, the finding that nearly three-quarters of patients taking daily oral ART were willing to switch suggests a substantial unmet need for alternative treatment delivery options in urban African settings.

A contrasting pattern was reported by Toska et al in a South African study where only about 12% of adolescents and young adults initially preferred injectables [20]. This difference may reflect competing priorities across age groups. Adults in our cohort often viewed a bi-monthly injection as relief from treatment fatigue and pill burden, whereas younger people may perceive rigid clinic appointments as a threat to autonomy and a potential source of unintended disclosure.

Acceptability, therefore, is not a fixed biomedical measure. It is shaped by age, lifestyle, and social context. Importantly, the South African study also found that preference for injectables increased markedly among youth who faced multiple adherence barriers, reaching 66%. This aligns closely with our findings: in Model 4, adherence challenges independently doubled the odds of accepting LAI-ART (aOR = 2.154). Across different populations in sub-Saharan Africa, difficulty maintaining daily oral therapy appears to be a consistent driver of interest in long-acting alternatives.

Our qualitative findings add an important layer of interpretation. A high acceptability percentage does not mean patients are unconditionally ready to transition to injectables. Rather, many participants expressed cautious optimism. They welcomed the possibility of reducing daily pill-taking, but remained anxious about side effects, long-term commitment, and the practical realities of injection schedules.

Several focus group participants specifically requested a “trial period” to see how their bodies would respond before committing to long-term treatment. This helps explain why some participants in the quantitative survey said they did not yet have enough information to make a definitive decision.

The motivations underlying interest in LAI-ART also differed from those commonly reported in high-income settings. In North America and Western Europe, long-acting injectables are often valued for convenience, travel flexibility, and relief from the daily reminder of living with HIV [7–13]. In our study, as well as in research among female sex workers in Tanzania and the Dominican Republic, motivations were more deeply tied to social protection and survival [16,26]. Participants described oral pill bottles as visible symbols that could threaten employment, housing, education, or family relationships. For many patients in Nairobi, avoiding disclosure was not simply about convenience; it was about preserving dignity, safety, and social stability.

This broader social context also helps explain why the crude difference in acceptability between Kenyatta National Hospital (KNH) and Aga Khan University Hospital (AKUH) disappeared after multivariable adjustment. Initially, patients at KNH appeared more likely to accept LAI-ART. But after accounting for differences in age and gender distribution, hospital site was no longer significant. Patients in both settings shared similar concerns about the inflexibility of injection schedules. Oral ART allows patients to travel with extra medication, but injectable therapy requires timely clinic attendance. Fear of missing appointments while traveling or living away from the clinic was common across both public and private sector patients, suggesting that logistical concerns transcend institutional differences.

Another key finding was the strong role of information access. General prior awareness of LAI-ART nearly tripled the adjusted odds of willingness to switch. More specifically, obtaining information from online sources independently doubled the odds of acceptability, even after adjusting for age. This challenges the assumption that digital health information mainly influences younger patients. In our cohort, online searching appeared to offer a private and low-risk way for individuals to learn about new treatment options without exposing themselves to stigma or unwanted disclosure within clinic or family settings.

Finally, although patients struggling with daily adherence were especially motivated to switch to LAI-ART, implementation must be approached carefully. Evidence from high-income country rollouts shows that some patients discontinue long-acting regimens because of injection-site reactions or other adverse events [17,18]. In sub-Saharan Africa, the consequences of interrupted injectable therapy may be particularly serious because of the prolonged pharmacokinetic “tail” of these drugs, during which sub-therapeutic concentrations can persist for months after a missed dose [27]. Missing scheduled injections may therefore increase the risk of developing NNRTI and INSTI resistance mutations. This is especially important in Kenya, where NNRTI resistance is already a recognized programmatic concern [28]. For that reason, LAI-ART should not be viewed simply as a solution to oral non-adherence. Successful implementation will require strong system-level support, including appointment reminders, decentralized delivery options, reliable follow-up systems, and proactive tracing of patients who miss visits. Without these safeguards, the same adherence challenges that drive interest in injectables could also undermine their long-term effectiveness.

### Strengths and limitations

This study has several notable strengths. It is among the first mixed-methods analyses of LAI-ART acceptability in Kenya, combining quantitative and qualitative data from both public and private tertiary facilities. Integrating the quantitative survey data with focus group discussions allowed us to see that high numerical acceptability actually represented cautious optimism rather than full readiness, providing a much deeper understanding of patient readiness than either method could achieve alone. In addition, the relatively large sample strengthens generalizability to similar urban populations.

However, our findings should be considered alongside some limitations First, the study was conducted exclusively at two tertiary hospitals in Nairobi, meaning the results may not fully represent patients seeking care in rural or lower-level facilities where system constraints differ. Second, because LAI-ART is not yet widely available in routine care in Kenya, reported willingness to switch is based on a hypothetical scenario and may not fully predict future uptake once available. Third, as a cross-sectional study, we captured patient perspectives at a single point in time, meaning we cannot account for how attitudes might change as patients get more exposure to the treatment or as national rollouts begin. Lastly, although our models successfully identified key predictors of interest, social desirability bias cannot be completely ruled out during the in-person surveys and focus groups.

## Conclusion

In summary, while LAI-ART is highly acceptable, uptake will depend on raising awareness, addressing stigma, building trust, and ensuring system preparedness. Tailoring implementation to different patient populations and facility contexts will be key to realizing its promise as a transformative option for HIV care in sub-Saharan Africa.

## Supporting information

S1 Fig Conceptual Framework for Acceptability of LAI-ART using the Consolidated Framework for Implementation Research. (DOCX)

S1 Table Supporting tables. (DOCX)

S1 File Dataset. (XLSX)
