# Supplementary material for: “The injection gives freedom” - An exploration of long-acting injectable HIV treatment acceptance among patients seeking care in two Nairobi tertiary hospitals
Source: PLoS One. 2026 Sep 30;21(9):e0320713. doi: 10.1371/journal.pone.0320713 (PMC13626336; doi:10.1371/journal.pone.0320713)
Supplement: Supplementary file 1 [file pone.0320713.s007.pdf]

Letter of Response to the Reviewers

Journal: PLOS ONE

Manuscript ID: PONE-D-25-09653R2

Title: “The injection gives freedom” - An exploration of long-acting injectable HIV treatment acceptance among patients seeking care in two Nairobi tertiary hospitals

Dear Dr. Ilse Bloom and the PLOS ONE Editorial Board,

Thank you for the constructive and thoughtful feedback on our manuscript. We are grateful to the editorial team, the statistical advisory board member, and the peer reviewers for their valuable guidance. We have carefully addressed all concerns raised by the Editor and the reviewers.

In response to the key recommendations from the statistical adviser and Reviewer #4, we substantially revised our quantitative analysis strategy. Specifically, we replaced the original modelling approach with an exposure-specific hierarchical multivariable framework that appropriately accounts for baseline confounding by age, gender, and facility site while minimizing the risk of mediator and overadjustment bias. A detailed point-by-point response to each comment is provided below.

Response to Additional Editor Comments

Comment 1: Study Design Paragraph 1

Expunge the first paragraph of the study design, as this paragraph repeats the ethical statement.

Response:

We agree and have removed the repetitive opening paragraph from the "Study Design" subsection. Ethical considerations are now presented exclusively within the dedicated "Ethical Statement" section, improving both clarity and structure while eliminating redundancy.

Comment 2: Study Design Paragraph 3

Expunge the 3rd paragraph of the study design, and move the different issues reported in the paragraph to the appropriate sub-sections of the methods section.

Response:

We have removed the third paragraph from the "Study Design" section and redistributed its contents to the relevant methodological subsections. Information relating to data collection instruments, prior validation, and electronic data capture has been incorporated into the "Data Collection Procedures" subsection. Clarifications regarding data independence and qualitative thematic mapping have been relocated to the "Quantitative Analysis" and "Qualitative Analysis" subsections, respectively.

Comments 3 & 7: Quantitative and Multivariable Regression Re-modelling

The authors should expunge the statement related to bivariate logistic regression... Additionally, the authors should follow the standard reporting format for the results of multivariate logistic regression.

Response:

We greatly appreciate this statistical correction and have comprehensively revised both our terminology and reporting approach.

First, we have replaced the term *bivariate logistic regression* with the correct term, *univariable logistic regression*, when referring to models containing a single predictor variable.

Second, we have separated the reporting of unadjusted and adjusted associations in line with accepted reporting standards. Crude odds ratios (cORs), 95% confidence intervals, and univariable p-values are now reported within the screening analyses presented in Supplementary Table 2.

Finally, Table 4 has been reformatted to present only adjusted odds ratios (aORs), corresponding 95% confidence intervals, and exact p-values from the final exposure-specific multivariable models.

Comment 4: Splitting Descriptive Tables

Split sociodemographic factors and medical history into two tables.

Response:

We have divided the original combined table into two separate tables: Table 1 (Sociodemographic Characteristics) and Table 2 (Medical History).

Comment 5: Table Formatting Optimization

Avoid repeating '%' in all cells by denoting frequency (n) and percentage (%) in the title of the tables.

Response:

We have removed redundant percentage symbols from individual table cells throughout the manuscript. Frequencies and percentages are now clearly identified within the table headers and column titles.

Comment 6: Bivariate Scope Expansion

Table 3 shows the results of your cross-tabulation of acceptability with 12 variables... The authors should include all 24 variables...

Response:

We appreciate the request for greater transparency. All variables collected during the study are now formally evaluated in the screening analyses. The revised text discusses crude associations across demographic, social, and clinical domains. To maintain readability within the main manuscript, the complete cross-tabulation of all screened variables is provided in Supplementary Table S2.

Comments 8 & 9: Discussion Structure Optimization

The authors should start the discussion with a paragraph summarizing the objective of the study, the key findings and sets the tone for the discussion...

Response:

We have completely restructured the Discussion section to follow the recommended format. The section now opens with a concise summary of the study objective and principal findings before progressing through each major theme in sequence. These include overall acceptability, the theme of cautious optimism identified in the qualitative data, the attenuation of site-level differences after adjustment, the influence of prior awareness, and the role of online information sources.

Comment 10: Discussion Integration

The authors should integrate any new findings into the discussion when they have addressed comment number 6...

Response:

We have incorporated the expanded univariable and multivariable findings throughout the Discussion. Additional sections now examine how treatment complexity, adherence challenges, and other clinical factors influence patient preferences, with these quantitative findings contextualized using qualitative narratives from the focus group discussions.

Response to Reviewers' Comments

Reviewer #4

Major Comment 1: Addressing Overadjustment and Mediator Bias Through Exposure-Specific Modelling

Response:

We are grateful for this insightful epidemiological critique, which substantially strengthened the manuscript. We agree that the original saturated multivariable model risked introducing both overadjustment and mediator bias by simultaneously including variables that may lie along the causal pathway.

In response, we abandoned the single-model approach and adopted an exposure-specific hierarchical modelling framework. Four separate multivariable models were developed, each designed to estimate the total effect of a specific exposure while adjusting only for appropriate baseline confounders, namely age, gender, and facility site.

Model 1 examined the effect of facility site while controlling for age and gender. The previously observed institutional difference was no longer statistically significant (aOR = 1.188; 95% CI: 0.659–2.141; $p = 0.567$), indicating that the crude association was largely attributable to demographic differences between sites.

Model 2 assessed the effect of prior awareness of LAI-ART and demonstrated a strong independent association with acceptability (aOR = 2.851; 95% CI: 1.742–4.666; $p < 0.001$).

Model 3 evaluated specific information channels and showed that obtaining information from online sources independently doubled the odds of treatment acceptability (aOR = 2.246; 95% CI: 1.303–3.873; $p = 0.004$).

Model 4 focused on clinical experiences and treatment-related factors, demonstrating that adherence challenges and difficulties maintaining daily oral therapy were independently associated with increased willingness to switch to LAI-ART (aOR = 2.154; 95% CI: 1.266–3.664; $p = 0.005$).

Major Comment 2: Viral Suppression Target Population

Response:

This is an important clinical consideration. Our study was designed as a cross-sectional survey of routine outpatient attendees rather than a clinical trial or cohort study. Consequently, plasma HIV-RNA results were not systematically collected as part of the study dataset. However, participants were required to be on stable ART, and routine viral load monitoring is standard practice under Kenyan national treatment guidelines.

Minor Comment 1: Discontinuation Rates and NNRTI/INSTI Resistance

Response:

We agree that this is a critical issue for future implementation. The Introduction and Discussion sections have been expanded to include evidence from real-world LAI-ART programs documenting discontinuation due to injection-site reactions and treatment fatigue. We also discuss the implications of the prolonged pharmacokinetic tail associated with long-acting formulations and the potential for treatment interruptions to promote the emergence of NNRTI and INSTI resistance mutations, particularly in resource-limited settings.

Minor Comment 2: Regression Nomenclature

Response:

Thank you for highlighting this issue. We have reviewed the manuscript in its entirety and replaced all instances of "bivariate logistic regression" with the correct term, "univariable logistic regression."

Minor Comment 3: Abstract Clarification

Response:

We agree that the original wording could be misinterpreted. To improve clarity, we have replaced the term "education" with "patient-centered communication campaigns and increased awareness of LAI-ART." This more accurately reflects the informational and awareness-related factors identified in our findings rather than formal educational attainment.

We believe these rigorous enhancements fully resolve all peer-review comments and make our paper highly suitable for publication in *PLOS ONE*. We look forward to hearing your final decision.

Sincerely,

Duncan Nyukuri

Dr. Duncan Nyukuri Wekesa

Email: dnyukuri@gmail.com
