# Supplementary material for: “The injection gives freedom” - An exploration of long-acting injectable HIV treatment acceptance among patients seeking care in two Nairobi tertiary hospitals
Source: PLoS One. 2026 Sep 30;21(9):e0320713. doi: 10.1371/journal.pone.0320713 (PMC13626336; doi:10.1371/journal.pone.0320713)
Supplement: Supplementary file 2 [file pone.0320713.s001.docx]

S1 Fig. Conceptual Framework for Acceptability of LAI-ART using the Consolidated Framework for Implementation Research
