# Supplementary material for: “The injection gives freedom” - An exploration of long-acting injectable HIV treatment acceptance among patients seeking care in two Nairobi tertiary hospitals
Source: PLoS One. 2026 Sep 30;21(9):e0320713. doi: 10.1371/journal.pone.0320713 (PMC13626336; doi:10.1371/journal.pone.0320713)
Supplement: Supplementary file 3 [file pone.0320713.s002.docx]

**Good Reporting of a Mixed Methods Study checklist**

| **Good Reporting of A Mixed Methods Study (GRAMMS) checklist Guideline** | **Section: page** |
| --- | --- |
| Describe the justification for using a mixed methods approach to the research question | Methods- study design: pg. 6 |
| Describe the design in terms of the purpose, priority and sequence of methods | Methods- Study design: pg. 7 |
| Describe each method in terms of sampling, data collection and analysis | Methods - Sampling and recruitment - pg. 8  Methods - Data collection procedures: pg. 8  Methods - Analysis: pg. 9-10 |
| Describe where integration has occurred, how it has occurred and who has participated in it | Methods - Integration of findings: pg. 11 |
| Describe any limitation of one method associated with the present of the other method | Strengths and limitations pg. 35 |
| Describe any insights gained from mixing or integrating methods | Strengths and limitations pg. 35 |

**Cross-tabulation of Acceptability of LAI-ART by Participant Characteristics**

| **Characteristic** | **Category** | **No (n=99)** | **Yes (n=257)** | **Crude Odds Ratio (cOR; 95% CI)** | **p-value** |
| --- | --- | --- | --- | --- | --- |
| Site | AKUH *(Ref)*  KNH | 30  69 | 51  206 | 1.00  **1.75 (1.04–2.95)** | **0.035** |
| Age Group (Years) | 20–29 *(Ref)*  30–39  40–49  50–59  60+ | *  20  38  26  9 | *  73  77  70  12 | 1.00  0.88 (0.32–2.42)  0.49 (0.19–1.27)  0.65 (0.24–1.73)  0.32 (0.10–1.07) | 0.141 |
| Gender | Female *(Ref)*  Male | 52  47 | 158  99 | 1.00  0.69 (0.43–1.11) | 0.124 |
| Marital Status | Single *(Ref)*  Married  Separated / Divorced  Widowed | 22  60  6  11 | 65  158  22  12 | 1.00  0.89 (0.51–1.57)  1.24 (0.45–3.41)  0.37 (0.14–0.96) | 0.142 |
| Living Arrangement | Alone *(Ref)*  As a Couple  With Family | 28  11  60 | 59  14  184 | 1.00  0.60 (0.23–1.57)  1.46 (0.87–2.45) | 0.069 |
| Has Children | No *(Ref)*  Yes | 18  81 | 47  210 | 1.00  0.99 (0.54–1.83) | 0.981 |
| Employment Status | No *(Ref)*  Yes | 22  77 | 43  214 | 1.00  1.42 (0.81–2.51) | 0.230 |
| Cigarette Use | No *(Ref)*  Yes | 93  6 | 243  14 | 1.00  0.89 (0.33–2.41) | 0.822 |
| Alcohol Use | No *(Ref)*  Yes | 78  21 | 204  53 | 1.00  0.97 (0.55–1.68) | 0.902 |
| Recreational Drug Use | No *(Ref)*  Yes | 99  0 | 253  4 | 1.00 | 0.579^a^ |
| Hypertension | No *(Ref)*  Yes | 83  16 | 214  43 | 1.00  1.04 (0.56–1.95) | 0.897 |
| Diabetes Mellitus | No *(Ref)*  Yes | 94  5 | 239  18 | 1.00  1.42 (0.51–3.94) | 0.502 |
| High Cholesterol | No *(Ref)*  Yes | 95  4 | 242  15 | 1.00  1.47 (0.48–4.53) | 0.499 |
| Psychiatric / Depression | No *(Ref)*  Yes | 92  7 | 230  27 | 1.00  1.54 (0.65–3.67) | 0.323 |
| Other Medical History | No *(Ref)*  Yes | 86  13 | 227  30 | 1.00  0.87 (0.44–1.75) | 0.705 |
| Duration since Infection | 1–5 years *(Ref)*  < 1 year  5–10 years  > 10 years  Undisclosed | 22  7  28  34  8 | 72  20  51  92  22 | 1.00  0.87 (0.32–2.38)  0.56 (0.27–1.14)  0.83 (0.45–1.52)  0.84 (0.31–2.28) | 0.507 |
| Duration on ART | 1–5 years *(Ref)*  < 1 year  5–10 years  > 10 years  Undisclosed | 25  7  25  35  7 | 75  20  53  85  24 | 1.00  0.95 (0.36–2.54)  0.71 (0.35–1.41)  0.81 (0.44–1.48)  1.14 (0.43–3.03) | 0.796 |
| Current Dosing Frequency | Once Daily *(Ref)*  More than Once Daily | 94  5 | 247  10 | 1.00  0.76 (0.25–2.31) | 0.571^a^ |
| Treatment Perception | Complicated *(Ref)*  Simple | 3  96 | 27  230 | 1.00  **0.27 (0.08–0.90)** | **0.023** |
| Adverse Effects (Side Effects) | No *(Ref)*  Yes | 65  34 | 137  120 | 1.00  **1.67 (1.03–2.71)** | **0.035** |
| Treatment Tolerance Barriers | No *(Ref)*  Yes | 11  88 | 38  219 | 1.00  0.72 (0.35–1.48) | 0.367 |
| Missed ART Doses | No *(Ref)*  Yes | 64  35 | 109  148 | 1.00  **2.48 (1.53–4.03)** | **< 0.001** |
| Care Access (Visit Frequency) | Every 2–3 Months *(Ref)*  Every 6 Months | 70  29 | 172  85 | 1.00  1.19 (0.73–1.96) | 0.493 |
| Partner Aware of Status | No *(Ref)*  Yes | 37  62 | 104  153 | 1.00  0.88 (0.55–1.41) | 0.593 |
| Family Aware of Status | No *(Ref)*  Yes | 50  49 | 141  116 | 1.00  0.84 (0.53–1.33) | 0.460 |
| Friends Aware of Status | No *(Ref)*  Yes | 89  10 | 223  34 | 1.00  1.36 (0.65–2.85) | 0.422 |
| Colleagues Aware of Status | No *(Ref)*  Yes | 90  9 | 243  14 | 1.00  0.58 (0.24–1.37) | 0.210 |
| Change due to Effectiveness | No *(Ref)*  Yes | 85  14 | 218  39 | 1.00  1.09 (0.57–2.08) | 0.806 |
| Change due to Tolerance | No *(Ref)*  Yes | 83  16 | 221  36 | 1.00  0.85 (0.45–1.58) | 0.606 |
| Change due to Adherence | No *(Ref)*  Yes | 97  2 | 252  5 | 1.00  0.96 (0.18–5.02) | 1.000^a^ |
| Prior Aware of LAI-ART | No *(Ref)*  Yes | 51  48 | 73  184 | 1.00  **2.67 (1.68–4.26)** | **< 0.001** |
| Info Source: Provider | No *(Ref)*  Yes | 65  34 | 158  99 | 1.00  1.20 (0.74–1.94) | 0.465 |
| Info Source: Support Group | No *(Ref)*  Yes | 86  13 | 200  57 | 1.00  1.89 (0.99–3.62) | 0.054 |
| Info Source: Online Platforms | No *(Ref)*  Yes | 74  25 | 154  103 | 1.00  **1.98 (1.18–3.33)** | **0.009** |
| Info Source: Other Channels | No *(Ref)*  Yes | 99  0 | 254  3 | 1.00 | 0.563^a^ |

^.^

* Cell suppressed (N < 5) to protect participant anonymity

^a^ P-value calculated using Fisher's Exact Test due to expected cell counts < 5.

To optimize the dataset for bivariate analysis and address sparse cell counts, variables were operationalized and collapsed as follows:

- **ART Dosing Frequency:** "Twice a day" and "More than Twice a day" were merged into **"More than once a day."**
- **ART Treatment Perception:** "Unmanageable" and "complicated" were grouped under **"Complicated."**
- **Adverse Effects from ART:** Recoded into a binary format (**"Yes"** = minor/major effects; **"No"** = never).
- **Missed ART Doses:** Recoded into a binary format (**"Yes"** = sometimes/regularly; **"No"** = never).
- **HIV Status Disclosure (Partner, Family, Friends, Colleagues):** "No" and "I prefer not to say" were merged under **"No."**
- **Prior ART Regimen Change (Effectiveness, Tolerance, Adherence):** "No" and "I don't know" were merged under **"No."**

**Preferences, Advantages & Disadvantages of LAI-ART (Acceptors Only)**

| **Characteristic** | **Category** | **KNH 163** | **AKUH 50** | **Combined 213** |
| --- | --- | --- | --- | --- |
| Preferred duration | 1 month | 47 (28.8% | 17 (34.0%) | 64 (30.0%) |
|  | 2 months | 42 (25.8) | 38 (76.0%) | 80 (37.6%) |
|  | 3 months | 52 (31.9%) | 18 (36.0%) | 70 (32.9%) |
|  | 4 months | 2 (1.2%) | 1 (2.0%) | 3 (1.4%) |
|  | 6 months | 85 (52.1%) | 7 (14.0%) | 92 (43.2%) |
|  | 12 months | 8 (4.9%) | 2 (4.0%) | 10 (4.7%) |
| **Characteristic** | **Category** | **KNH** | **AKUH** | **Combined** |
| Advantages of LAI-ART | Stop daily treatment | 160 (77.7%) | 40 (78.4%) | 200 (77.8%) |
|  | Ensure effectiveness | 20 (9.7%) | 20 (39.2%) | 40 (15.6%) |
|  | Not forget medication | 106 (51.5%) | 37 (72.5%) | 143 (55.6%) |
|  | Hide from others | 88 (42.7%) | 29 (56.9%) | 117 (45.5%) |
|  | Forget disease | 90 (43.7%) | 5 (9.8%) | 95 (37.0%) |
|  | Not think daily | 123 (59.7%) | 6 (11.8%) | 129 (50.2%) |
|  | Other | 7 (3.4%) | 0 (0.0%) | 7 (2.7%) |
| **Characteristic** | **Category** | **KNH** | **AKUH** | **Combined** |
| Disadvantage | Lose freedom | 1 (0.5%) | 0 (0.0%) | 1 (0.4%) |
|  | Side effects | 130 (63.1%) | 45 (88.2%) | 175 (68.1%) |
|  | Injections | 65 (31.6%) | 7 (13.7%) | 72 (28.0%) |
|  | Guinea pig | 42 (20.4%) | 4 (7.8%) | 46 (17.9%) |
|  | Other | 4 (1.9%) | 1 (2.0%) | 5 (1.9%) |
