# Supplementary material for: “The injection gives freedom” - An exploration of long-acting injectable HIV treatment acceptance among patients seeking care in two Nairobi tertiary hospitals
Source: PLoS One. 2026 Sep 30;21(9):e0320713. doi: 10.1371/journal.pone.0320713 (PMC13626336; doi:10.1371/journal.pone.0320713)
Supplement: Supplementary file 4 [file pone.0320713.s005.docx]

Date: 23 August 2025

To:

Dr. Daniel Chukwuemeka Ogbuabor, Ph.D., M.D.

Academic Editor

PLOS ONE

**RE: Submission of Revised Manuscript, PONE-D-25-09653 - "The injection gives freedom” - An exploration of long-acting injectable HIV treatment acceptance among patients seeking care in two Nairobi tertiary hospitals"**

Dear Dr Ogbuabor,

Thank you for the opportunity to revise our manuscript. We are also grateful to the reviewers for their time and for providing such insightful and constructive feedback. We have found the comments to be extremely helpful and believe that addressing them has substantially strengthened our paper.

We have performed a complete and thorough re-analysis of our data, which has yielded a more nuanced and robust set of findings. The manuscript has been extensively revised to incorporate these new results and to address every point raised by you and the reviewers.

Below, we provide a point-by-point response to each of the comments.

**Response to Editor's Comments**

**Editor Comment 1:** *“Please include your full ethics statement in the ‘Methods’ section of your manuscript file. In your statement, please include the full name of the IRB or ethics committee who approved or waived your study, as well as whether or not you obtained informed written or verbal consent.”*

**Response:** We have now included a dedicated subsection titled “Ethical Statement” within the “Materials and Methods” section. This statement provides the full names of the approving ethics committees (Kenyatta National Hospital-University of Nairobi ERC and the Institutional Research and Ethics Committee (IREC) of Aga Khan University) and confirms that all participants provided written informed consent.

**Editor Comment 2:** *“In addition to the reviewers' comments, the authors should extend the inferential analysis of acceptability to a regression analysis. The authors should consider dichotomizing the acceptability (outcome) variable... The demographic characteristics, knowledge, and awareness variables will be the independent variables (predictors).”*

**Response:** We have performed a comprehensive re-analysis as requested. The “Data Analysis” subsection of our Methods now details the full statistical plan. We conducted bivariate and multivariate logistic regression analyses to identify factors associated with LAI-ART acceptability for the combined cohort and stratified by hospital site. The full results of this new analysis, including both Unadjusted and Adjusted Odds Ratios, are presented in the revised Results section and the new Table 3. These findings are now a central part of our rewritten Discussion section.

**Response to Reviewer 1 Comments**

**Comment 1:** *“Please be explicit in indicating that this was NOT a clinical trial.”*

**Response:** We have now explicitly stated this in the Study Design subsection of the Methods: “Importantly, this was not a clinical trial; no medications were administered.”

**Comments 2 & 3:** *“Introduction: I would be clearer in stating that most available data on LAI-ART is from the United States and Western Europe and hence data from Eastern and Southern Africa the epicenter of the HIV epidemic is needed. I would cite studies conducted on LAI-ART from Uganda, South Africa etc.”*

**Response:** We have revised the Introduction to more clearly state that much of the pivotal data comes from high-income countries, and we have strengthened the landscape analysis of the growing literature from SSA, including citations from studies conducted in Uganda and South Africa.

**Comment 4:** *“Introduction: I would include statistics on the state of adherence to ART. Which sub-populations have adherence constraints in Kenya?”*

**Response:** We included adherence data, highlighting that adolescents and young adults in Kenya often show lower adherence due to stigma and transition challenges.

**Comments 5** *CFIR framework is only mentioned, not described.*

**Response:** We expanded the Methods to explain how CFIR informed both the development of tools and the coding framework for qualitative analysis.

**Comment 6:** *Methods: FGD details are scant. Why 3 FGDs? Were gender/age dynamics considered?*

**Response:** We have also provided more detail on the FGDs, including the purposive sampling strategy and the fact that we continued until thematic saturation was reached.

**Comments 7** *Missed opportunity to show synergy between qualitative and quantitative data.*

**Response:** We strengthened the Integration of findings section, explicitly using triangulation to highlight convergence, divergence, and complementarity between survey and FGD findings.

**Comments 8:** *Data analysis for quantitative vs qualitative muddled. Separate them.*

**Response:** The Data analysis section now has separate, clear subsections for the quantitative and qualitative analysis plans.

**Comments 9:** *Wrong heading on page 13: qualitative results labelled as quantitative.*

**Response:** The incorrect heading has been removed.

**Comments 10 & 11:** *“Presentation of qualitative illustrative quotes: it always more preferable to have indented quotes as stand-alone. I would include a table of the emergent themes/ sub-themes as informed by CFIR framework.”*

**Response:** We have reformatted all qualitative quotes as indented, stand-alone block quotes. We have also added a new Table 4 in the Results section, sub-section “Qualitative findings and integrated analysis”, which summarizes the emergent qualitative themes as informed by the CFIR framework, as requested.

**Response to Reviewer 2 Comments**

**Comment 1:** *“The scientific contribution of the study is not clearly defined... The manuscript does not clarify what is new or which specific gap it is addressing.”*

**Response:** We have revised the final paragraph of the Introduction to more clearly articulate the study's specific contribution: examining the acceptability of LAI-ART in a general population of PLHIV (as opposed to specific high-risk groups) and doing so across both the public and private tertiary healthcare sectors in Kenya, a comparison that is not well-represented in the existing literature.

**Comment 2:** *“CFIR Framework: The article mentions the CFIR, but does not demonstrate how the qualitative analysis was truly anchored in this framework.”*

**Response:** We have strengthened the link to the CFIR throughout the manuscript. The **Methods** section now clarifies its role in guiding the study, and a new Table 4 in the Results explicitly structures our qualitative themes according to the CFIR domains.

**Comment 3** *Probability sampling: not well explained.*

**Response:** Methods now specify that survey participants were recruited using systematic random sampling from clinic logs. Sample size was calculated using Cochran’s formula (95% CI, 5% margin, prevalence 66% from Toska et al).

**Comment 4:** *Questionnaire validation not described.*

**Response:** Added that the survey was adapted from a validated tool by Simoni et al., used in Tanzania and Dominican Republic.

**Comment 5:** *Chi-square analysis assumptions unclear; p-values not adjusted.*

**Response:** We now explicitly state that Chi-square assumptions were checked, Fisher’s exact used when cell counts <5, and p-values reported are unadjusted but interpreted cautiously.

Confidence intervals are reported for regression.

**Comments 6 & 7:** *Comments on qualitative methodological rigor (sample size justification, coding, reflexivity.).*

**Response:** We have expanded the qualitative methods subsection to provide much greater detail on our process. We now include justification for our sample size (thematic saturation), a description of the moderator’s experience, details on our dual-coder process to ensure reliability, and a note on using peer debriefing to validate our interpretations.

**Comment 8:** *“Integration Between Methods (Mixed Methods): This is the manuscript’s weakest point... The quantitative and qualitative results are entirely compartmentalized.”*

**Response:** The Results section has been completely restructured. It now features a dedicated subsection, “Qualitative Findings: An Integrated Analysis,” which systematically weaves the two datasets together. We explicitly present quantitative findings and immediately use qualitative quotes to explain them. This section is now framed around analyzing the convergence and divergence between the two data types.

**Comment 9:** *“The manuscript’s discussion is overly focused on repeating descriptive data without delving into contradictions or tensions... Moreover, it lacks reflections on the implications these findings have for public health practices in Kenya, especially considering differences between the public system... and the private sector.”*

**Response:** The Discussion section has been completely rewritten based on our new, more robust analysis. It no longer repeats results but focuses on interpreting the key findings, with a particular emphasis on the newly identified statistically significant difference in acceptability between the KNH (public) and AKUH (private) sites. We now discuss the tensions and paradoxes revealed by the integrated results and provide a much stronger section on the concrete implications for public health and implementation strategies in Kenya, considering the different healthcare settings.

**Comment 10:** *“Finally, there is excessive repetition of concepts... The text requires greater objectivity and synthesis.”*

**Response:** We have carefully edited the entire manuscript to improve clarity, objectivity, and synthesis, and to eliminate repetitive phrasing.

We thank you and the reviewers again for your valuable guidance. We believe the manuscript is now substantially improved and hope that it is now suitable for publication in [Journal Name].

Sincerely,

Duncan Nyukuri (on behalf of all co-authors)

Consultant Physician and Infectious Disease Specialist, Kenyatta National Hospital

[dnyukuri@gmail.com](mailto:dnyukuri@gmail.com), +254 720977887
