# Supplementary material for: “The injection gives freedom” - An exploration of long-acting injectable HIV treatment acceptance among patients seeking care in two Nairobi tertiary hospitals
Source: PLoS One. 2026 Sep 30;21(9):e0320713. doi: 10.1371/journal.pone.0320713 (PMC13626336; doi:10.1371/journal.pone.0320713)
Supplement: Supplementary file 5 [file pone.0320713.s006.docx]

# Response to Reviewers

Date: 07 October 8, 2025

To:

Dr. Daniel Chukwuemeka Ogbuabor, Ph.D., M.D.

Academic Editor

PLOS ONE

**Manuscript Title:** ““The injection gives freedom” – An exploration of long-acting injectable HIV treatment acceptance among patients seeking care in two Nairobi tertiary hospitals”, PONE-D-25-09653

Dear Dr Ogbuabor,
We thank you for the opportunity to revise and resubmit our manuscript and for the insightful feedback provided by both the reviewers and the editorial team. We have carefully addressed all journal requirements and editor comments, leading to significant improvement in methodological rigor, reporting quality, and manuscript structure.
Below we outline our responses in two parts: (1) Journal Requirements and (2) Specific Editor Comments.

## Part 1: Response to Journal Requirements

### 1. Review of Recommended Citations

We reviewed all previously suggested publications and evaluated their relevance to our study context. Only those that were directly relevant and contributed substantively to the evidence base were cited.
- We incorporated the study suggested by one of the initial reviewers:
 O’Cathain A, Murphy E, Nicholl J. The quality of mixed methods studies in health services research. J Health Serv Res Policy. 2008 Apr; 13(2): 92–98. [Available from: https://pubmed.ncbi.nlm.nih.gov/18416914/]. This foundational paper strengthens the methodological justification of our mixed-methods design by providing an accepted framework for assessing quality and integration.
- To operationalize this, we added a new supplementary table — “S1 Table. Good Reporting of a Mixed Methods Study (GRAMMS) Checklist” — mapping our procedures and integration points against GRAMMS domains for transparency.

### 2. Reference List Accuracy and Completeness

A full audit of the reference list was performed to ensure accuracy and consistency.
- References 2, 6, 14, 15, and 16 from the first version, previously redacted, were reinstated because they provide essential global evidence on LAI-ART efficacy, satisfaction, and implementation barriers.
- References 11–13 and 18–21 from the first version were removed, as the Discussion was substantially rewritten per reviewer guidance and the sections citing them were deleted or modified.
- All remaining references were cross-checked via PubMed and Google Scholar for current status; none were found to be retracted.

## Part 2: Response to Editor’s Specific Comments

### Comment 1. Age group

Implemented as advised. All tables and text now categorize participants ≥ 60 years under a single group.

**Comment 2. Marital status**

Categories merged into “Divorced/Separated/Not stated.”

**Comment 3. Employment status**

Implemented. The merged category “Not employed” now appears in Table 1 and corresponding results text.

**Comment 4. Bivariate comparisons in Tables 1 and 2**

All p-values and statistical comparisons were removed from Tables 1 and 2, which now display descriptive summaries only. Inferential analyses are relocated to a dedicated Table 3.

**Comment 5. Cross-tabulation and selection of variables for logistic regression**

A new Table 3: Cross-tabulation of Acceptability of LAI-ART by Participant Characteristics has been created, reporting Chi-square or Fisher’s exact p-values. Independent variables with p < 0.05 were entered into a multivariate binary logistic-regression model, the results of which are shown in Table 4.

**Comment 6. Presentation of binary logistic regression results**

The regression output has been expanded. Table 4 now displays both significant and non-significant predictors with Crude (OR) and Adjusted (OR) estimates, 95% CIs, and p-values. The narrative under Results → Predictors of Acceptability and the corresponding Discussion paragraph were updated accordingly.

**Comment 7. Revise Results and Discussion in light of methodological changes**

The Results and Discussion were revised to reflect corrected analyses and variable recoding.

### Additional Note on Tables

To enhance clarity, most tables previously in the Supplementary Material have been moved into the main manuscript text:
- Former Supplementary Tables 1 and 2 are now Tables 1 and 2 (Demographics, Awareness & Acceptability).
- Cross-tabulation and regression outputs appear as Tables 3 and 4.
- Only S1 Table (GRAMMS Checklist) and S2 Table (Preferences, Advantages & Disadvantages of LAI-ART (Acceptors Only)) remain as supplementary materials.

These changes ensure that key quantitative findings are presented prominently within the article body for improved readability and transparency.

We appreciate the editor’s and reviewers’ constructive feedback, which has substantially strengthened the manuscript. We believe that the current revision now meets all methodological, analytical, and reporting requirements for publication.

Sincerely,

Dr. Duncan Wekesa Nyukuri, MBCHB, MMed, Fellow (ID)
Corresponding Author
On behalf of all co-authors
