# Supplementary material for: “The injection gives freedom” - An exploration of long-acting injectable HIV treatment acceptance among patients seeking care in two Nairobi tertiary hospitals
Source: PLoS One. 2026 Sep 30;21(9):e0320713. doi: 10.1371/journal.pone.0320713 (PMC13626336; doi:10.1371/journal.pone.0320713)
Supplement: Peer review history

# Peer review: Decision Letter 0

**DOI:** 10.1371/journal.pone.0320713.r001

**Authors:** Daniel Ogbuabor

9 Jul 2025

Dear Dr. Nyukuri,

Thank you for submitting your manuscript to PLOS ONE. After careful consideration, we feel that it has merit but does not fully meet PLOS ONE’s publication criteria as it currently stands. Therefore, we invite you to submit a revised version of the manuscript that addresses the points raised during the review process.

Please submit your revised manuscript by Aug 23 2025 11:59PM. If you will need more time than this to complete your revisions, please reply to this message or contact the journal office at plosone@plos.org. When you're ready to submit your revision, log on to https://www.editorialmanager.com/pone/ and select the 'Submissions Needing Revision' folder to locate your manuscript file.

- A rebuttal letter that responds to each point raised by the academic editor and reviewer(s). You should upload this letter as a separate file labeled 'Response to Reviewers'.
- A marked-up copy of your manuscript that highlights changes made to the original version. You should upload this as a separate file labeled 'Revised Manuscript with Track Changes'.
- An unmarked version of your revised paper without tracked changes. You should upload this as a separate file labeled 'Manuscript'.

If you would like to make changes to your financial disclosure, please include your updated statement in your cover letter. Guidelines for resubmitting your figure files are available below the reviewer comments at the end of this letter.

If applicable, we recommend that you deposit your laboratory protocols in protocols.io to enhance the reproducibility of your results. Protocols.io assigns your protocol its own identifier (DOI) so that it can be cited independently in the future. For instructions see: https://journals.plos.org/plosone/s/submission-guidelines#loc-laboratory-protocols. Additionally, PLOS ONE offers an option for publishing peer-reviewed Lab Protocol articles, which describe protocols hosted on protocols.io. Read more information on sharing protocols at https://plos.org/protocols?utm_medium=editorial-email&utm_source=authorletters&utm_campaign=protocols.

We look forward to receiving your revised manuscript.

Kind regards,

Daniel Chukwuemeka Ogbuabor, Ph.D., M.D.

Academic Editor

PLOS ONE

Journal Requirements:

When submitting your revision, we need you to address these additional requirements.

1. Please ensure that your manuscript meets PLOS ONE's style requirements, including those for file naming. The PLOS ONE style templates can be found at

https://journals.plos.org/plosone/s/file?id=wjVg/PLOSOne_formatting_sample_main_body.pdf  and

https://journals.plos.org/plosone/s/file?id=ba62/PLOSOne_formatting_sample_title_authors_affiliations.pdf

2. Please include your full ethics statement in the ‘Methods’ section of your manuscript file. In your statement, please include the full name of the IRB or ethics committee who approved or waived your study, as well as whether or not you obtained informed written or verbal consent. If consent was waived for your study, please include this information in your statement as well.

Additional Editor Comments:

In addition to the reviewers' comments, the authors should extend the inferential analysis of acceptability to a regression analysis. The authors should consider dichotomizing the acceptability (outcome) variable into "1 = Yes" and "0 = No, I do not have enough information, and I am not sure." The demographic characteristics, knowledge, and awareness variables will be the independent variables (predictors).

[Note: HTML markup is below. Please do not edit.]

Reviewers' comments:

Reviewer's Responses to Questions

**Comments to the Author**

1. Is the manuscript technically sound, and do the data support the conclusions?

Reviewer #1: Yes

Reviewer #2: No

**********

2. Has the statistical analysis been performed appropriately and rigorously?

Reviewer #1: Yes

Reviewer #2: No

**********

3. Have the authors made all data underlying the findings in their manuscript fully available?

The PLOS Data policy

Reviewer #1: No

Reviewer #2: No

**********

4. Is the manuscript presented in an intelligible fashion and written in standard English?

Reviewer #1: Yes

Reviewer #2: Yes

**********

Reviewer #1: The focus by the authors on long acting injectable HIV treatment is important and presents a novel alternative to oral HIV treatment. Most data on the subject has been from North America and Western Europe. Data from Africa is sparse hence this study is welcome.

I have a few comments that I hope can help strengthen the current manuscript.

1. Please be explicit in indicating that this was NOT a clinical trial.

2. Introduction: I would be clearer in stating that most available data on LAI-ART is from the United States and Western Europe and hence data from Eastern and Southern Africa the epicentre of the HIV epidemic is needed.

3. Introduction: I would cite studies conducted on LAI-ART from Uganda, South Africa etc. There is insufficient landscape analysis of previous studies on LAI-ART even within SSA.

4. Introduction: I would include statistics on the state of adherence to ART. Which sub-populations have adherence constraints in Kenya? How about adolescents and LAI-ART?

5. Methods: The CFIR framework is only mentioned and not described. How did it help the authors?

6. Methods: the information on FGDs is scanty and needs to be fleshed out more. Why six FGDs? Did you consider gender, age group dynamics, marital status etc?

7. There is a missed opportunity of synergies between the qualitative and quantitative arms of the study. Please reflect on this.

8. Data analysis: please seperate the analysis plans for the quantitative and qualitative arms of the study. They muddled up in the current draft see a model/ benchmark from Kathryn Church (2015) on a study on integration of HIV and family planning published in PLOS ONE.

9. Page 13: the heading says Quantitative results when actually these are qualitative studies.

10. Presentation of qualitative illustrative quotes: it always more preferable to have indented quotes as stand-alone.

11.I would include a table of the emergent themes/ sub-themes as informed by CFIR framework.

Reviewer #2: The manuscript explores the acceptability of long-acting injectable antiretroviral therapy (LAI-ART) among people living with HIV (PLHIV) in Nairobi. The topic is highly relevant given the context of HIV in Sub-Saharan Africa and the advances in treatment with long-acting regimens. However, despite the relevance and merit of the topic, the manuscript presents substantial shortcomings in methodological rigor in both the quantitative and qualitative components, as well as significant weaknesses in the integration between methods.

Below are my comments:

1. The scientific contribution of the study is not clearly defined. The existing literature already includes similar studies (including in Kenya) on the acceptability of LAI-ART. The manuscript does not clarify what is new or which specific gap it is addressing.

2. CFIR Framework: The article mentions the CFIR, but does not demonstrate how the qualitative analysis was truly anchored in this framework—it appears more as a superficial citation than a genuine analytical foundation.

3. The manuscript claims to have employed “probability sampling” for the quantitative study, yet it does not provide:

a. A detailed method of random selection (e.g., simple random, systematic).

b. The formula or parameters used for sample size calculation.

c. A justification of statistical power, even though inferential tests were used.

4. The quantitative questionnaire is not provided as an appendix, and the text does not indicate whether it was previously validated, subjected to pre-testing/piloting, or based on previously validated scales to measure constructs such as acceptability, perception of stigma, or adherence.

5. The use of chi-square tests is reported, but there is no information about: checking assumptions (e.g. expected cell counts ≥ 5); adjustments for multiple comparisons; or confidence intervals for proportions or odds ratios. Several p-values are reported, but without clarity on whether they are adjusted, which increases the risk of false positives (Type I error).

6. The manuscript does not present fundamental assumptions for the validity of the qualitative findings, including:

a. Criteria for determining the sample size in focus groups.

b. Justification of theoretical saturation (it is not mentioned whether saturation was achieved).

c. Detailed description of the focus group discussion guides.

d. The experience and positionality of the moderator beyond merely being “trained.”

e. Reflexivity (how the researchers’ positions may have influenced the responses).

f. Processes of member checking (validation of findings by participants).

g. Details on coding (units of analysis, examples of codes).

7. The qualitative analysis is described as “thematic” but without demonstrating how categories were constructed (inductive or deductive); examples of how quotations were translated into codes and themes; agreement between coders (level of agreement or Kappa statistic), or the relationship between the qualitative data and the CFIR beyond a mere mention of its domains.

8. Integration Between Methods (Mixed Methods): This is the manuscript’s weakest point:

The article is described as a mixed-methods study, but:

It presents no explicit strategy for integrating the data (e.g. triangulation, side-by-side comparison, joint display tables).

The quantitative and qualitative results are entirely compartmentalized.

There is no analysis of convergence or divergence between the two data sets.

It is not explained how the qualitative findings could explain or deepen the quantitative findings.

→ This violates basic principles of mixed-methods studies (e.g. Good Reporting of A Mixed Methods Study – GRAMMS).

9. The manuscript’s discussion is overly focused on repeating descriptive data without delving into contradictions or tensions between findings, nor exploring the meaning of these results in the context of practical implementation of LAI-ART. Moreover, it lacks reflections on the implications these findings have for public health practices in Kenya, especially considering differences between the public system, represented by Kenyatta National Hospital (KNH), and the private sector, such as Aga Khan University Hospital. The statements about “high acceptability” also seem somewhat exaggerated, since about 20% of participants still expressed uncertainty or insufficient information, and most preferred longer intervals between injections than those offered by current regimens. Additionally, there are significant structural barriers—including issues of access, cost, and logistics—that may compromise the practical feasibility of large-scale implementation of injectable therapy.

10. Finally, there is excessive repetition of concepts (e.g. “LAI-ART reduces stigma”) throughout the discussion. The text requires greater objectivity and synthesis, particularly in the qualitative sections.

**********

what does this mean?). If published, this will include your full peer review and any attached files.

If you choose “no”, your identity will remain anonymous but your review may still be made public.

**Do you want your identity to be public for this peer review?**  For information about this choice, including consent withdrawal, please see our Privacy Policy

Reviewer #1: No

Reviewer #2: No

**********

[NOTE: If reviewer comments were submitted as an attachment file, they will be attached to this email and accessible via the submission site. Please log into your account, locate the manuscript record, and check for the action link "View Attachments". If this link does not appear, there are no attachment files.]

While revising your submission, please upload your figure files to the Preflight Analysis and Conversion Engine (PACE) digital diagnostic tool, https://pacev2.apexcovantage.com/. PACE helps ensure that figures meet PLOS requirements. To use PACE, you must first register as a user. Registration is free. Then, login and navigate to the UPLOAD tab, where you will find detailed instructions on how to use the tool. If you encounter any issues or have any questions when using PACE, please email PLOS at figures@plos.org. Please note that Supporting Information files do not need this step.

# Author comment: Author response to Decision Letter 1

**DOI:** 10.1371/journal.pone.0320713.r002

25 Aug 2025

Date: 23 August 2025

To:

Dr. Daniel Chukwuemeka Ogbuabor, Ph.D., M.D.

Academic Editor

PLOS ONE

RE: Submission of Revised Manuscript, PONE-D-25-09653 - "The injection gives freedom"—An exploration of long-acting injectable HIV treatment acceptance among patients seeking care in two Nairobi tertiary hospitals"

Dear Dr Ogbuabor,

Thank you for the opportunity to revise our manuscript. We are also grateful to the reviewers for their time and for providing such insightful and constructive feedback. We have found the comments to be extremely helpful and believe that addressing them has substantially strengthened our paper.

We have performed a complete and thorough reanalysis of our data, which has yielded a more nuanced and robust set of findings. The manuscript has been extensively revised to incorporate these new results and to address every point raised by you and the reviewers.

Below, we provide a point-by-point response to each of the comments.

Response to Editor's Comments

Editor Comment 1: “Please include your full ethics statement in the ‘Methods’ section of your manuscript file. In your statement, please include the full name of the IRB or ethics committee who approved or waived your study, as well as whether or not you obtained informed written or verbal consent.”

Response: We have now included a dedicated subsection titled “Ethical Statement” within the “Materials and Methods” section. This statement provides the full names of the approving ethics committees (Kenyatta National Hospital-University of Nairobi ERC and the Institutional Research and Ethics Committee (IREC) of Aga Khan University) and confirms that all participants provided written informed consent.

Editor Comment 2: “In addition to the reviewers' comments, the authors should extend the inferential analysis of acceptability to a regression analysis. The authors should consider dichotomizing the acceptability (outcome) variable... The demographic characteristics, knowledge, and awareness variables will be the independent variables (predictors).”

Response: We have performed a comprehensive re-analysis as requested. The “Data Analysis” subsection of our Methods now details the full statistical plan. We conducted bivariate and multivariate logistic regression analyses to identify factors associated with LAI-ART acceptability for the combined cohort and stratified by hospital site. The full results of this new analysis, including both Unadjusted and Adjusted Odds Ratios, are presented in the revised Results section and the new Table 3. These findings are now a central part of our rewritten Discussion section.

Response to Reviewer 1 Comments

Comment 1: “Please be explicit in indicating that this was NOT a clinical trial.”

Response: We have now explicitly stated this in the Study Design subsection of the Methods: “Importantly, this was not a clinical trial; no medications were administered.”

Comments 2 & 3: “Introduction: I would be clearer in stating that most available data on LAI-ART is from the United States and Western Europe and hence data from Eastern and Southern Africa the epicenter of the HIV epidemic is needed. I would cite studies conducted on LAI-ART from Uganda, South Africa etc.”

Response: We have revised the Introduction to more clearly state that much of the pivotal data comes from high-income countries, and we have strengthened the landscape analysis of the growing literature from SSA, including citations from studies conducted in Uganda and South Africa.

Comment 4: “Introduction: I would include statistics on the state of adherence to ART. Which sub-populations have adherence constraints in Kenya?”

Response: We included adherence data, highlighting that adolescents and young adults in Kenya often show lower adherence due to stigma and transition challenges.

Comments 5 CFIR framework is only mentioned, not described.

Response: We expanded the Methods to explain how CFIR informed both the development of tools and the coding framework for qualitative analysis.

Comment 6: Methods: FGD details are scant. Why 3 FGDs? Were gender/age dynamics considered?

Response: We have also provided more detail on the FGDs, including the purposive sampling strategy and the fact that we continued until thematic saturation was reached.

Comments 7 Missed opportunity to show synergy between qualitative and quantitative data.

Response: We strengthened the Integration of findings section, explicitly using triangulation to highlight convergence, divergence, and complementarity between survey and FGD findings.

Comments 8: Data analysis for quantitative vs. qualitative is muddled. Separate them.

Response: The Data analysis section now has separate, clear subsections for the quantitative and qualitative analysis plans.

Comments 9: Wrong heading on page 13: qualitative results labelled as quantitative.

Response: The incorrect heading has been removed.

Comments 10 & 11: “Presentation of qualitative illustrative quotes: it always more preferable to have indented quotes as stand-alone. I would include a table of the emergent themes/ sub-themes as informed by CFIR framework.”

Response: We have reformatted all qualitative quotes as indented, stand-alone block quotes. We have also added a new Table 4 in the Results section, sub-section “Qualitative findings and integrated analysis”, which summarizes the emergent qualitative themes as informed by the CFIR framework, as requested.

Response to Reviewer 2 Comments

Comment 1: “The scientific contribution of the study is not clearly defined... The manuscript does not clarify what is new or which specific gap it is addressing.”

Response: We have revised the final paragraph of the Introduction to more clearly articulate the study's specific contribution: examining the acceptability of LAI-ART in a general population of PLHIV (as opposed to specific high-risk groups) and doing so across both the public and private tertiary healthcare sectors in Kenya, a comparison that is not well-represented in the existing literature.

Comment 2: “CFIR Framework: The article mentions the CFIR, but does not demonstrate how the qualitative analysis was truly anchored in this framework.”

Response: We have strengthened the link to the CFIR throughout the manuscript. The Methods section now clarifies its role in guiding the study, and a new Table 4 in the Results explicitly structures our qualitative themes according to the CFIR domains.

Comment 3 Probability sampling: not well explained.

Response: Methods now specify that survey participants were recruited using systematic random sampling from clinic logs. Sample size was calculated using Cochran’s formula (95% CI, 5% margin, prevalence 66% from Toska et al).

Comment 4: Questionnaire validation not described.

Response: Added that the survey was adapted from a validated tool by Simoni et al., used in Tanzania and the Dominican Republic.

Comment 5: Chi-square analysis assumptions unclear; p-values not adjusted.

Response: We now explicitly state that Chi-square assumptions were checked, Fisher’s exact used when cell counts <5, and p-values reported are unadjusted but interpreted cautiously.

Confidence intervals are reported for regression.

Comments 6 & 7: Comments on qualitative methodological rigor (sample size justification, coding, reflexivity.).

Response: We have expanded the qualitative methods subsection to provide much greater detail on our process. We now include justification for our sample size (thematic saturation), a description of the moderator’s experience, details on our dual-coder process to ensure reliability, and a note on using peer debriefing to validate our interpretations.

Comment 8: “Integration Between Methods (Mixed Methods): This is the manuscript’s weakest point... The quantitative and qualitative results are entirely compartmentalized.”

Response: The Results section has been completely restructured. It now features a dedicated subsection, “Qualitative Findings: An Integrated Analysis,” which systematically weaves the two datasets together. We explicitly present quantitative findings and immediately use qualitative quotes to explain them. This section is now framed around analyzing the convergence and divergence between the two data types.

Comment 9: “The manuscript’s discussion is overly focused on repeating descriptive data without delving into contradictions or tensions... Moreover, it lacks reflections on the implications these findings have for public health practices in Kenya, especially considering differences between the public system... and the private sector.”

Response: The Discussion section has been completely rewritten based on our new, more robust analysis. It no longer repeats results but focuses on interpreting the key findings, with a particular emphasis on the newly identified statistically significant difference in acceptability between the KNH (public) and AKUH (private) sites. We now discuss the tensions and paradoxes revealed by the integrated results and provide a much stronger section on the concrete implications for public health and implementation strategies in Kenya, considering the different healthcare settings.

Comment 10: “Finally, there is excessive repetition of concepts... The text requires greater objectivity and synthesis.”

Response: We have carefully edited the entire manuscript to improve clarity, objectivity, and synthesis and to eliminate repetitive phrasing.

We thank you and the reviewers again for your valuable guidance. We believe the manuscript is now substantially improved and hope that it is now suitable for publication in [Journal Name].

Sincerely,

Duncan Nyukuri (on behalf of all co-authors)

Attachment Submitted filename: Response to Reviewers.docx

# Editor report: Decision Letter 1

**DOI:** 10.1371/journal.pone.0320713.r003

**Authors:** Daniel Ogbuabor

10 Sep 2025

Dear Dr. Nyukuri,

- address the methodological changes and revised analyses advised.

plosone@plos.org. When you're ready to submit your revision, log on to https://www.editorialmanager.com/pone/ and select the 'Submissions Needing Revision' folder to locate your manuscript file.

- A rebuttal letter that responds to each point raised by the academic editor and reviewer(s). You should upload this letter as a separate file labeled 'Response to Reviewers'.
- A marked-up copy of your manuscript that highlights changes made to the original version. You should upload this as a separate file labeled 'Revised Manuscript with Track Changes'.
- An unmarked version of your revised paper without tracked changes. You should upload this as a separate file labeled 'Manuscript'.

If applicable, we recommend that you deposit your laboratory protocols in protocols.io to enhance the reproducibility of your results. Protocols.io assigns your protocol its own identifier (DOI) so that it can be cited independently in the future. For instructions see: https://journals.plos.org/plosone/s/submission-guidelines#loc-laboratory-protocols. Additionally, PLOS ONE offers an option for publishing peer-reviewed Lab Protocol articles, which describe protocols hosted on protocols.io. Read more information on sharing protocols at https://plos.org/protocols?utm_medium=editorial-email&utm_source=authorletters&utm_campaign=protocols.

We look forward to receiving your revised manuscript.

Kind regards,

Daniel Chukwuemeka Ogbuabor, Ph.D., M.D.

Academic Editor

PLOS ONE

Journal Requirements:

If the reviewer comments include a recommendation to cite specific previously published works, please review and evaluate these publications to determine whether they are relevant and should be cited. There is no requirement to cite these works unless the editor has indicated otherwise.

Please review your reference list to ensure that it is complete and correct. If you have cited papers that have been retracted, please include the rationale for doing so in the manuscript text, or remove these references and replace them with relevant current references. Any changes to the reference list should be mentioned in the rebuttal letter that accompanies your revised manuscript. If you need to cite a retracted article, indicate the article’s retracted status in the References list and also include a citation and full reference for the retraction notice.

Additional Editor Comments:

The revised manuscript is a significant improvement on the original version. Nonetheless, there are few methodological issues to resolve to get it to a publishable level.

1. Age group - I suggest merging 60-69 and 70-79 years into one category labelled 60 years and above.

2. Marital staus - merge 'divorced, separated and I prefer not say' into one category.

3. Employment status - Merge 'No' and 'I prefer not say group.'

4. The comparison (bivariate analyses) in Tables 1 and 2 are unnecessary, since the tables are meant to show descriptive statistics; and not inferential! If the significant variables from the two tables informed the multivariate logistic regression reported in Table 4, then the analysis was wrong. Please see my comment 5 for the correct statistical approach.

5. The author should conduct and report a cross-tabulation of acceptability of LAI (dependent variable) and independent variables (study site, age group, gender, marital status, living arrangements, has children, employment status, awareness of LAI and sources of information) with inferential analysis (Chi-square statistics and p-value). The independent variables that are statistically significant (p-value < 0.05) should be included in the multivariate binary logistic regression. The independent variables with significant odd ratios should be modelled to identify their adjusted odd ratios.

6. It is good practice to report the binary logistic regression results that include significant and insignificant results as well as the adjusted odd ratios for the significant variables.

7. Revise the results and discussion sections in the light of the methological changes and revised analyses.

[Note: HTML markup is below. Please do not edit.]

[NOTE: If reviewer comments were submitted as an attachment file, they will be attached to this email and accessible via the submission site. Please log into your account, locate the manuscript record, and check for the action link "View Attachments". If this link does not appear, there are no attachment files.]

While revising your submission, please upload your figure files to the Preflight Analysis and Conversion Engine (PACE) digital diagnostic tool, https://pacev2.apexcovantage.com/. PACE helps ensure that figures meet PLOS requirements. To use PACE, you must first register as a user. Registration is free. Then, login and navigate to the UPLOAD tab, where you will find detailed instructions on how to use the tool. If you encounter any issues or have any questions when using PACE, please email PLOS at figures@plos.org. Please note that Supporting Information files do not need this step.

# Author comment: Author response to Decision Letter 2

**DOI:** 10.1371/journal.pone.0320713.r004

9 Oct 2025

Response to Editor’s Specific Comments

Comment 1. Age group

Implemented as advised. All tables and text now categorize participants ≥ 60 years under a single group.

Comment 2. Marital status

Categories merged into “Divorced/Separated/Not stated.”

Comment 3. Employment status

Implemented. The merged category “Not employed” now appears in Table 1 and corresponding results text.

Comment 4. Bivariate comparisons in Tables 1 and 2

All p-values and statistical comparisons were removed from Tables 1 and 2, which now display descriptive summaries only. Inferential analyses are relocated to a dedicated Table 3.

Comment 5. Cross-tabulation and selection of variables for logistic regression

A new Table 3: Cross-tabulation of Acceptability of LAI-ART by Participant Characteristics has been created, reporting Chi-square or Fisher’s exact p-values. Independent variables with p < 0.05 were entered into a multivariate binary logistic-regression model, the results of which are shown in Table 4.

Comment 6. Presentation of binary logistic regression results

The regression output has been expanded. Table 4 now displays both significant and non-significant predictors with Crude (OR) and Adjusted (OR) estimates, 95% CIs, and p-values. The narrative under Results → Predictors of Acceptability and the corresponding Discussion paragraph were updated accordingly.

Comment 7. Revise Results and Discussion in light of methodological changes

The Results and Discussion were revised to reflect corrected analyses and variable recoding.

Additional Note on Tables

To enhance clarity, most tables previously in the Supplementary Material have been moved into the main manuscript text:

- Former Supplementary Tables 1 and 2 are now Tables 1 and 2 (Demographics, Awareness & Acceptability).

- Cross-tabulation and regression outputs appear as Tables 3 and 4.

- Only S1 Table (GRAMMS Checklist) and S2 Table (Preferences, Advantages & Disadvantages of LAI-ART (Acceptors Only)) remain as supplementary materials.

Attachment Submitted filename: Response to Reviewers v2.docx

# Peer review: Decision Letter 2

**DOI:** 10.1371/journal.pone.0320713.r005

**Authors:** Ilse Bloom

30 Apr 2026

Dear Dr. Nyukuri,

Thank you for submitting your manuscript to PLOS ONE. After careful consideration, we feel that it has merit but does not fully meet PLOS ONE’s publication criteria as it currently stands. Therefore, we invite you to submit a revised version of the manuscript that addresses the points raised during the review process.

==============================

The manuscript has been further evaluated by two reviewers, and their comments are available below.

In the previous round of review the Academic Editor had some comments on the statistical analysis. As they are no longer available, we have consulted a member of our statistical advisory board. This reviewer has raised major concerns regarding the statistical analysis conducted.

Could you please carefully revise the manuscript to address all comments raised?

==============================

Please submit your revised manuscript by Jun 14 2026 11:59PM. If you will need more time than this to complete your revisions, please reply to this message or contact the journal office at plosone@plos.org. When you're ready to submit your revision, log on to https://www.editorialmanager.com/pone/ and select the 'Submissions Needing Revision' folder to locate your manuscript file.

- A letter that responds to each point raised by the academic editor and reviewer(s). You should upload this letter as a separate file labeled 'Response to Reviewers'.
- A marked-up copy of your manuscript that highlights changes made to the original version. You should upload this as a separate file labeled 'Revised Manuscript with Track Changes'.
- An unmarked version of your revised paper without tracked changes. You should upload this as a separate file labeled 'Manuscript'.

If you would like to make changes to your financial disclosure, please include your updated statement in your cover letter. Guidelines for resubmitting your figure files are available below the reviewer comments at the end of this letter.

If applicable, we recommend that you deposit your laboratory protocols in protocols.io to enhance the reproducibility of your results. Protocols.io assigns your protocol its own identifier (DOI) so that it can be cited independently in the future. For instructions see: https://journals.plos.org/plosone/s/submission-guidelines#loc-laboratory-protocols. Additionally, PLOS ONE offers an option for publishing peer-reviewed Lab Protocol articles, which describe protocols hosted on protocols.io. Read more information on sharing protocols at https://plos.org/protocols?utm_medium=editorial-email&utm_source=authorletters&utm_campaign=protocols.

As the corresponding author, your ORCID iD is verified in the submission system and will appear in the published article. PLOS supports the use of ORCID, and we encourage all coauthors to register for an ORCID iD and use it as well. Please encourage your coauthors to verify their ORCID iD within the submission system before final acceptance, as unverified ORCID iDs will not appear in the published article. *Only*  the individual author can complete the verification step; PLOS staff *cannot*  verify ORCID iDs on behalf of authors.

We look forward to receiving your revised manuscript.

Kind regards,

Ilse Bloom

Staff Editor

PLOS One

**Journal Requirements:**

If the reviewer comments include a recommendation to cite specific previously published works, please review and evaluate these publications to determine whether they are relevant and should be cited. There is no requirement to cite these works unless the editor has indicated otherwise.

**Additional Editor Comments:**

Study design.

1.     Expunge the first paragraph of the study design, as this paragraph repeats the ethical statement.

2.     Expunge the 3rd paragraph of the study design, and move the different issues reported in the paragraph to the appropriate sub-sections of the methods’ section.

Methods

3.     The authors should expunge the statement related to bivariate logistic regression in the multivariable analysis under the quantitative analysis component of data analysis. To generate crude and adjusted odd ratios, you run multivariate binary regression in two stages. In the first stage, all significant results from the cross-tabulation (bivariate analysis) should be moved into the multivariate binary regression model to generate the crude odd ratios. All the variables with p-value less than 0.05 in the initial multivariate binary regression should be moved into the stage 2 multivariate binary regression model to generate the adjusted odd ratios.

Results

4.     Split sociodemographic factors and medical history into two tables.

5.     In all Tables that report frequencies and percentages, avoid repeating ‘%’ in all cells by denoting frequency (n) and percentage (%) in the title of the tables.

6.     Table 3 shows the results of your cross-tabulation of acceptability with 12 variables (bivariate analysis). However, Table 1 has 24 variables (SDF and medical history). The authors should include all 24 variables in the cross-tabulation; otherwise, justify their exclusion, or explain why they collected data that were not useful.

7.     See my comment number 3 in the methods’ section about multivariate binary logistic regression. Additionally, the authors should follow the standard reporting format for the results of multivariate logistic regression. Examples abound in the literature. It is not appropriate to report COR and AOR as done in Table 4.

Discussion

8.     The authors should start the discussion with a paragraph summarizing the objective of the study, the key findings and sets the tone for the discussion. For example: “The objective of the study was to determine the acceptability of LAI-ART and its associated factors in Kenya. The study found high acceptability driven high pill burden, adherence, and privacy. Yet, the finding of cautious optimism is concerning due to side-effects, treatment rigidity, and logistic challenges. The predictors of acceptability were site, level of awareness, and online resources of information. These significant findings warrant further exploration.”

9.     The authors should then proceed to discuss the one finding at a time – high acceptability, cautious optimism, influence of site, awareness, and online sources.

10.   The authors should integrate any new findings into the discussion when they have addressed the comments number 6 (inclusion of more variables in the inferential analysis).

[Note: HTML markup is below. Please do not edit.]

Reviewers' comments:

Reviewer's Responses to Questions

**Comments to the Author**

Reviewer #3: All comments have been addressed

Reviewer #4: (No Response)

**********

2. Is the manuscript technically sound, and do the data support the conclusions?

Reviewer #3: Yes

Reviewer #4: Partly

**********

3. Has the statistical analysis been performed appropriately and rigorously?

Reviewer #3: Yes

Reviewer #4: No

**********

4. Have the authors made all data underlying the findings in their manuscript fully available?

The PLOS Data policy

Reviewer #3: Yes

Reviewer #4: Yes

**********

5. Is the manuscript presented in an intelligible fashion and written in standard English?

Reviewer #3: Yes

Reviewer #4: Yes

**********

**Reviewer #3:**  All editor and journal requirements have been comprehensively addressed. Age groups were consolidated, marital and employment categories merged, and bivariate comparisons removed from descriptive tables. New cross-tabulation (Table 3) and expanded regression (Table 4) tables were added with complete statistical outputs. Results and Discussion sections were revised accordingly. Key tables were moved from supplementary materials to the main text, and the GRAMMS checklist was added to enhance methodological transparency.

**Reviewer #4:**  This is an interesting survey study conducted at two tertiary hospitals in Nairobi, Kenya: a private hospital, the Aga Khan University Hospital (AKUH) and the largest public hospital in the same area Kenyatta National Hospital (KNH).

Data are somewhat informative as LAI-ART are and increasingly popular treatment option and there are not many similar studies in the low-income settings. However, I have a reservation regarding the quantitative analysis as well as other minor points. I am not an expert in qualitive research so I will not comment on those results or on those of the mixed analysis.

Major

1. Mine is one simple but fundamental critique. I do not particularly like the multivariable models that have been constructed. Essentially the authors constructed a model by including factors that were associated (i.e. p-value was <0.05) in univariable analysis. This approach has two major problems. Say we are interested in source of awareness of LAI-ART as the main exposure. Age is a potential confounder for this, as younger participants are more likely to have had the information from online sources and Table 3 also shows that younger age was also associated with higher rate of acceptability. Thus, a model which does not account for age is likely to over-estimate the OR for online resources (some of the effect may be explained by younger age). Same arguments apply for other potential confounders such as sex, etc. Conversely, the OR for the factor hospital in a regression model which also includes source of awareness of LAI-ART is difficult to interpret. Such a model (like the one shown in Table 4) can estimate at best what is the effect of site which is not mediated by the source of awareness type. Indeed, enrolment in AKUH seems to lead mainly to awareness due to online resources so that source of awareness is a potential intermediate in the relationship between hospital-site and rate of acceptability. It would be easier to have separate tables for each of the exposure of interest showing the total effect for all the exposures so they are directly comparable. In this example I suggest one Table for source of awareness as the exposure (showing unadjusted and adjusted OR from a model only including potential confounders of source of awareness: e.g. hospital, age, sex etc.) and separate tables for the total effect of hospital and for the total effect of awareness still controlling only for confounders. Thus, for example age is a confounder of both hospital and source of awareness while source of awareness is not a confounder for hospital (getting online information about LAI-ARI does not determine in which hospital the participant is treated), etc. What is the total effect of awareness (which was identified by the authors as a key predictor) after controlling for confounding (age, sex, etc.)?

2. The main inclusion criterion is that participants had to be on stable ART. However, the target population for LAI-ART would be people with current suppressed viral load. Do we know how many of the participants had elevated HIV-RNA despite ART?

Minor points

1. Introduction. “Long-acting injectable antiretroviral therapy (LAI-ART) offers a promising alternative to the daily pill regimen for treating HIV. Administered every two months, this innovative therapy could simplify treatment, improve patient adherence, and boost retention in care (1,2).” One key issue that was observed in people treated with LAI-ART in the western word -which is likely to occur also in low income settings- is the high rate of discontinuation because of intolerance at site of injection and long-term treatment fatigue. This discontinuations are also often associated with accumulation of NNRTI-resistance which will be an issue in Kenya. Suggest this is mentioned here and in the Discussion with appropriate references.

2. Nomenclature: Logistic regression model with a single outcome and a single covariate are called univariable model, not bivariate. Bivariate models are typically regression jointly modelling two outcomes.

3. Abstract. “These findings suggest that while LAI-ART is seen as promising, its uptake will depend on education, trust-building, and reliable delivery systems”. Assume “education” mainly refers to “prior awareness of LAI-ART” not level of education in general (e.g. university vs. elementary school) so it may need to be qualified.

**********

what does this mean?). If published, this will include your full peer review and any attached files.

If you choose “no”, your identity will remain anonymous but your review may still be made public.

**Do you want your identity to be public for this peer review?**  For information about this choice, including consent withdrawal, please see our Privacy Policy

Reviewer #3: No

Reviewer #4: No

**********

[NOTE: If reviewer comments were submitted as an attachment file, they will be attached to this email and accessible via the submission site. Please log into your account, locate the manuscript record, and check for the action link "View Attachments". If this link does not appear, there are no attachment files.]

To ensure your figures meet our technical requirements, please review our figure guidelines: https://journals.plos.org/plosone/s/figures

You may also use PLOS’s free figure tool, NAAS, to help you prepare publication quality figures: https://journals.plos.org/plosone/s/figures#loc-tools-for-figure-preparation.

NAAS will assess whether your figures meet our technical requirements by comparing each figure against our figure specifications.

# Author comment: Author response to Decision Letter 3

**DOI:** 10.1371/journal.pone.0320713.r006

29 Jun 2026

Dear Dr. Ilse Bloom and the PLOS ONE Editorial Board,

Thank you for the constructive and thoughtful feedback on our manuscript. We are grateful to the editorial team, the statistical advisory board member, and the peer reviewer for their valuable guidance. We have carefully addressed all concerns raised by the Editor and the reviewers.

In response to the key recommendations from the editor and Reviewer 4, we substantially revised our quantitative analysis strategy. Specifically, we replaced the original modelling approach with an exposure-specific hierarchical multivariable framework that appropriately accounts for baseline confounding by age, gender, and facility site while minimizing the risk of mediator and overadjustment bias. A detailed point-by-point response to each comment is provided below.

Response to Additional Editor Comments

Comment 1: Study Design Paragraph 1

Expunge the first paragraph of the study design, as this paragraph repeats the ethical statement.

Response:

We agree and have removed the repetitive opening paragraph from the "Study Design" subsection. Ethical considerations are now presented exclusively within the dedicated "Ethical Statement" section, improving both clarity and structure while eliminating redundancy.

Comment 2: Study Design Paragraph 3

Expunge the 3rd paragraph of the study design, and move the different issues reported in the paragraph to the appropriate sub-sections of the methods section.

Response:

We have removed the third paragraph from the "Study Design" section and redistributed its contents to the relevant methodological subsections. Information relating to data collection instruments, prior validation, and electronic data capture has been incorporated into the "Data Collection Procedures" subsection. Clarifications regarding data independence and qualitative thematic mapping have been relocated to the "Quantitative Analysis" and "Qualitative Analysis" subsections, respectively.

Comments 3 & 7: Quantitative and Multivariable Regression Re-modelling

The authors should expunge the statement related to bivariate logistic regression... Additionally, the authors should follow the standard reporting format for the results of multivariate logistic regression.

Response:

We greatly appreciate this statistical correction and have comprehensively revised both our terminology and reporting approach.

First, we have replaced the term bivariate logistic regression with the correct term, univariable logistic regression, when referring to models containing a single predictor variable.

Second, we have separated the reporting of unadjusted and adjusted associations in line with accepted reporting standards. Crude odds ratios (cORs), 95% confidence intervals, and univariable p-values are now reported within the screening analyses presented in Supplementary Table 2.

Finally, Table 4 has been reformatted to present only adjusted odds ratios (aORs), corresponding 95% confidence intervals, and exact p-values from the final exposure-specific multivariable models.

Comment 4: Splitting Descriptive Tables

Split sociodemographic factors and medical history into two tables.

Response:

We have divided the original combined table into two separate tables: Table 1 (Sociodemographic Characteristics) and Table 2 (Medical History).

Comment 5: Table Formatting Optimization

Avoid repeating '%' in all cells by denoting frequency (n) and percentage (%) in the title of the tables.

Response:

We have removed redundant percentage symbols from individual table cells throughout the manuscript. Frequencies and percentages are now clearly identified within the table headers and column titles.

Comment 6: Bivariate Scope Expansion

Table 3 shows the results of your cross-tabulation of acceptability with 12 variables... The authors should include all 24 variables...

Response:

We appreciate the request for greater transparency. All variables collected during the study are now formally evaluated in the screening analyses. The revised text discusses crude associations across demographic, social, and clinical domains. To maintain readability within the main manuscript, the complete cross-tabulation of all screened variables is provided in Supplementary Table S2.

Comments 8 & 9: Discussion Structure Optimization

The authors should start the discussion with a paragraph summarizing the objective of the study, the key findings and sets the tone for the discussion...

Response:

We have completely restructured the Discussion section to follow the recommended format. The section now opens with a concise summary of the study objective and principal findings before progressing through each major theme in sequence. These include overall acceptability, the theme of cautious optimism identified in the qualitative data, the attenuation of site-level differences after adjustment, the influence of prior awareness, and the role of online information sources.

Comment 10: Discussion Integration

The authors should integrate any new findings into the discussion when they have addressed comment number 6...

Response:

We have incorporated the expanded univariable and multivariable findings throughout the Discussion. Additional sections now examine how treatment complexity, adherence challenges, and other clinical factors influence patient preferences, with these quantitative findings contextualized using qualitative narratives from the focus group discussions.

Response to Reviewers' Comments

Reviewer 4

Major Comment 1: Addressing Overadjustment and Mediator Bias Through Exposure-Specific Modelling

Response:

We are grateful for this insightful epidemiological critique, which substantially strengthened the manuscript. We agree that the original saturated multivariable model risked introducing both overadjustment and mediator bias by simultaneously including variables that may lie along the causal pathway.

In response, we abandoned the single-model approach and adopted an exposure-specific hierarchical modelling framework. Four separate multivariable models were developed, each designed to estimate the total effect of a specific exposure while adjusting only for appropriate baseline confounders, namely age, gender, and facility site.

Model 1 examined the effect of facility site while controlling for age and gender. The previously observed institutional difference was no longer statistically significant (aOR = 1.188; 95% CI: 0.659–2.141; p = 0.567), indicating that the crude association was largely attributable to demographic differences between sites.

Model 2 assessed the effect of prior awareness of LAI-ART and demonstrated a strong independent association with acceptability (aOR = 2.851; 95% CI: 1.742–4.666; p < 0.001).

Model 3 evaluated specific information channels and showed that obtaining information from online sources independently doubled the odds of treatment acceptability (aOR = 2.246; 95% CI: 1.303–3.873; p = 0.004).

Model 4 focused on clinical experiences and treatment-related factors, demonstrating that adherence challenges and difficulties maintaining daily oral therapy were independently associated with increased willingness to switch to LAI-ART (aOR = 2.154; 95% CI: 1.266–3.664; p = 0.005).

Major Comment 2: Viral Suppression Target Population

Response:

This is an important clinical consideration. Our study was designed as a cross-sectional survey of routine outpatient attendees rather than a clinical trial or cohort study. Consequently, plasma HIV-RNA results were not systematically collected as part of the study dataset. However, participants were required to be on stable ART, and routine viral load monitoring is standard practice under Kenyan national treatment guidelines.

Minor Comment 1: Discontinuation Rates and NNRTI/INSTI Resistance

Response:

We agree that this is a critical issue for future implementation. The Introduction and Discussion sections have been expanded to include evidence from real-world LAI-ART programs documenting discontinuation due to injection-site reactions and treatment fatigue. We also discuss the implications of the prolonged pharmacokinetic tail associated with long-acting formulations and the potential for treatment interruptions to promote the emergence of NNRTI and INSTI resistance mutations, particularly in resource-limited settings.

Minor Comment 2: Regression Nomenclature

Response:

Thank you for highlighting this issue. We have reviewed the manuscript in its entirety and replaced all instances of "bivariate logistic regression" with the correct term, "univariable logistic regression."

Minor Comment 3: Abstract Clarification

Response:

We agree that the original wording could be misinterpreted. To improve clarity, we have replaced the term "education" with "patient-centered communication campaigns and increased awareness of LAI-ART." This more accurately reflects the informational and awareness-related factors identified in our findings rather than formal educational attainment.

We believe these rigorous enhancements fully resolve all peer-review comments and make our paper highly suitable for publication in PLOS ONE. We look forward to hearing your final decision.

Attachment Submitted filename: Letter of Response to the Reviewers V4.pdf

# Peer review: Decision Letter 3

**DOI:** 10.1371/journal.pone.0320713.r007

**Authors:** Habil Otanga

10 Sep 2026

<p>“The injection gives freedom”— An exploration of long-acting injectable HIV treatment acceptance among patients seeking care in two Nairobi tertiary hospitals

PONE-D-25-09653R3

Dear Dr. Duncan Nyukuri,

We’re pleased to inform you that your manuscript has been judged scientifically suitable for publication and will be formally accepted for publication once it meets all outstanding technical requirements.

Within one week, you’ll receive an e-mail detailing the required amendments. When these have been addressed, you’ll receive a formal acceptance letter and your manuscript will be scheduled for publication.

An invoice will be generated when your article is formally accepted. Please note, if your institution has a publishing partnership with PLOS and your article meets the relevant criteria, all or part of your publication costs will be covered. Please make sure your user information is up-to-date by logging into Editorial Manager at Editorial Manager® and clicking the ‘Update My Information' link at the top of the page. For questions related to billing, please contact billing support.

If your institution or institutions have a press office, please notify them about your upcoming paper to help maximize its impact. If they’ll be preparing press materials, please inform our press team as soon as possible -- no later than 48 hours after receiving the formal acceptance. Your manuscript will remain under strict press embargo until 2 pm Eastern Time on the date of publication. For more information, please contact onepress@plos.org.

Kind regards,

Habil Otanga, Ph.D

Academic Editor

PLOS One

Additional Editor Comments (optional):

I note that substantial revisions were done to the manuscript to improve its quality.

Reviewers' comments:

Reviewer's Responses to Questions

**Comments to the Author**

Reviewer #5: All comments have been addressed

**********

2. Is the manuscript technically sound, and do the data support the conclusions?

Reviewer #5: Yes

**********

3. Has the statistical analysis been performed appropriately and rigorously?

Reviewer #5: Yes

**********

4. Have the authors made all data underlying the findings in their manuscript fully available?

The PLOS Data policy

Reviewer #5: Yes

**********

5. Is the manuscript presented in an intelligible fashion and written in standard English?

Reviewer #5: Yes

**********

Reviewer #5: Following earlier recommendations, I note that authors substantially revised their quantitative analysis strategy. They replaced the original modelling approach with an exposure-specific hierarchical multivariable framework that appropriately accounts for baseline confounding by age, gender, and facility site while minimizing the risk of mediator and overadjustment bias.

**********

what does this mean?). If published, this will include your full peer review and any attached files.

If you choose “no”, your identity will remain anonymous but your review may still be made public.

**Do you want your identity to be public for this peer review?**  For information about this choice, including consent withdrawal, please see our Privacy Policy

Reviewer #5: No

**********

# Editor report: Acceptance letter

**DOI:** 10.1371/journal.pone.0320713.r008

**Authors:** Habil Otanga

PONE-D-25-09653R3

PLOS One

Dear Dr. Nyukuri,

I'm pleased to inform you that your manuscript has been deemed suitable for publication in PLOS One. Congratulations! Your manuscript is now being handed over to our production team.

At this stage, our production department will prepare your paper for publication. This includes ensuring the following:

* All references, tables, and figures are properly cited

* All relevant supporting information is included in the manuscript submission,

* There are no issues that prevent the paper from being properly typeset

You will receive further instructions from the production team, including instructions on how to review your proof when it is ready. Please keep in mind that we are working through a large volume of accepted articles, so please give us a few days to review your paper and let you know the next and final steps.

Lastly, if your institution or institutions have a press office, please let them know about your upcoming paper now to help maximize its impact. If they'll be preparing press materials, please inform our press team within the next 48 hours. Your manuscript will remain under strict press embargo until 2 pm Eastern Time on the date of publication. For more information, please contact onepress@plos.org.

You will receive an invoice from PLOS for your publication fee after your manuscript has reached the completed accept phase. If you receive an email requesting payment before acceptance or for any other service, this may be a phishing scheme. Learn how to identify phishing emails and protect your accounts at https://explore.plos.org/phishing.

If we can help with anything else, please email us at customercare@plos.org.

Thank you for submitting your work to PLOS One and supporting open access.

Kind regards,

PLOS One Editorial Office Staff

on behalf of

Dr. Habil Otanga

Academic Editor

PLOS One
